# Toll-like receptor 3 activation selectively reverses HIV latency in microglial cells

**DOI:** 10.1186/s12977-017-0335-8

**Published:** 2017-02-06

**Authors:** David Alvarez-Carbonell, Yoelvis Garcia-Mesa, Stephanie Milne, Biswajit Das, Curtis Dobrowolski, Roxana Rojas, Jonathan Karn

**Affiliations:** Department of Molecular Biology and Microbiology, Case Western Reserve University, 10900 Euclid Ave., SOM WRT 200, Cleveland, OH 44106 USA

**Keywords:** HIV latency, Toll-like receptors, TLR3, Microglial cells, HIV-associated neurocognitive disorders

## Abstract

**Background:**

Multiple toll-like receptors (TLRs) are expressed in cells of the monocytic lineage, including microglia, which constitute the major reservoir for human immunodeficiency virus (HIV) infection in the brain. We hypothesized that TLR receptor mediated responses to inflammatory conditions by microglial cells in the central nervous system (CNS) are able to induce latent HIV proviruses, and contribute to the etiology of HIV-associated neurocognitive disorders.

**Results:**

Newly developed human microglial cell lines (hµglia), obtained by immortalizing human primary microglia with simian virus-40 (SV40) large T antigen and the human telomerase reverse transcriptase, were used to generate latently infected cells using a single-round HIV virus carrying a green fluorescence protein reporter (hµglia/HIV, clones HC01 and HC69). Treatment of these cells with a panel of TLR ligands showed surprisingly that two potent TLR3 agonists, poly (I:C) and bacterial ribosomal RNA potently reactivated HIV in hμglia/HIV cells. LPS (TLR4 agonist), flagellin (TLR5 agonist), and FSL-1 (TLR6 agonist) reactivated HIV to a lesser extent, while Pam3CSK4 (TLR2/1 agonist) and HKLM (TLR2 agonist) only weakly reversed HIV latency in these cells. While agonists for TLR2/1, 4, 5 and 6 reactivated HIV through transient NF-κB induction, poly (I:C), the TLR3 agonist, did not activate NF-κB, and instead induced the virus by a previously unreported mechanism mediated by IRF3. The selective induction of IRF3 by poly (I:C) was confirmed by chromatin immunoprecipitation (ChIP) analysis. In comparison, in latently infected rat-derived microglial cells (hT-CHME-5/HIV, clone HC14), poly (I:C), LPS and flagellin were only partially active. The TLR response profile in human microglial cells is also distinct from that shown by latently infected monocyte cell lines (THP-1/HIV, clone HA3, U937/HIV, clone HUC5, and SC/HIV, clone HSCC4), where TLR2/1, 4, 5, 6 or 8, but not for TLR3, 7 or 9, reactivated HIV.

**Conclusions:**

TLR signaling, in particular TLR3 activation, can efficiently reactivate HIV transcription in infected microglia, but not in monocytes or T cells. The unique response profile of microglial cells to TLR3 is fundamental to understanding how the virus responds to continuous microbial exposure, especially during inflammatory episodes, that characterizes HIV infection in the CNS.

**Electronic supplementary material:**

The online version of this article (doi:10.1186/s12977-017-0335-8) contains supplementary material, which is available to authorized users.

## Background

Although highly-active anti-retroviral therapies (HAART) can reduce circulating virus to below the levels of detection, these regimens are unable to eliminate residual viral infections due to the creation of long-lived reservoirs of latently infected cells [1]. The best characterized of these reservoirs are the resting memory CD4^+^ T cells found in the peripheral circulation [2, 3]. However, additional cell types, including peripheral blood monocytes, dendritic cells and macrophages in the lymph node, and astrocytes, perivascular macrophages and microglial cells in the brain, can also be infected with HIV and can potentially contribute to viral persistence [4–8].

Unlike T cells, cells of the monocyte–macrophage lineage are partially resistant to HIV infection due to the activity of the SAMHD1 restriction factor [9]. Less than 1% of the HIV-1 DNA in the peripheral circulation is found in circulating monocytes. Nonetheless, there is increasing evidence that myeloid cells facilitate the dissemination of the virus and contribute to the persistence of viral reservoirs in the CNS [10]. It seems likely that perivascular macrophages initiate brain infection, which then spreads to resident microglial cell populations [10–12]. Analysis of the sequence and phenotypes of viruses recovered from the CNS demonstrates that HIV in HAND patients represents a distinct, macrophage tropic virus population [13–15]. It has been speculated that once infected, monocyte–macrophage lineage cells are more resistant to certain anti-retroviral drugs and the cytopathic and apoptotic effects of HIV than T cells, and therefore can harbor actively replicating viruses for longer periods even under conditions of effective suppression by HAART. Detection of viral escape mutations in the cerebrospinal fluid of individuals under HAART with undetectable viral presence in blood supports this hypothesis [16–18].

One of the main drivers of HIV infections, and the reason why there is chronic inflammation during HIV disease, is that damage to the gastrointestinal track leads to release of microbial products into the circulation [19]. These potent inducers of inflammatory responses are recognized by pattern recognition receptors, including the ten TLRs (TLR1–10), which are responsible for the innate recognition of viruses, bacteria, fungi, and parasites [20]. Monocytic cells characteristically express most, if not all, of the TLR family members, while T cells can only express a more limited set of these pattern recognition receptors. In response to the presence of pathogens invading the CNS, microglial cells activate their TLRs and initiate CNS innate and adaptive immune responses [21]. Excessive and/or chronic TLR activation of microglial cells in the brain [22] is believed to be directly responsible for a number of CNS diseases, including chronic HIV encephalitis, and the mounting pro-inflammatory reactions in response to HIV infections that lead to neurocognitive disorders [23, 24].

The signaling pathways following TLR activation are complex, and involve a series of protein adaptors (MyD88, TIRAP, TRIF, and TRAM) and downstream effectors (IRAKs, TRAF6, and TRAF3). In general, TLR signaling cascades derived from receptors 2/1, 4, 5, 6, and 8 eventually lead to the activation of the IKK complex and the translocation of the transcription factors NF-κB and AP-1 to the nucleus. Alternative pathways to this central signaling mechanism result in the production of type I interferons (IFNs) and IFN-inducible genes by activation of IRF7 (TLR7 and 9) and IRF3 (TLR3) [25]. Since reactivation of latent HIV proviruses can be potently stimulated by NF-κB in many cell types [26–29], TLR signaling can potentially lead to enhanced viral replication in cells that carry the appropriate receptors.

Even though TLR pathways have been relatively well characterized, investigations of TLR activation on HIV expression in various target cell types have been scarce, and largely limited to studies of transformed monocytic and T cell lines. Pomerantz et al. [30] initially reported that LPS, a bacterial TLR4 ligand, potently stimulated HIV-1 long terminal repeats (LTR) CAT constructs transfected into monocyte/macrophage-like cell lines, but not a T cell line. Subsequently, LPS-mediated activation in macrophages was linked to the activity of the PU.1 and NF-κB transcription factors [31–33], and the direct participation of TLR4 in mediating LPS-induced NF-κB and HIV-LTR activation was established [32, 33]. Later studies using the monocytic THP-1 cells showed that TLR2 ligands can also stimulate HIV transcription [33].

An inconsistent set of results has been obtained for the other TLR ligands. TLR9 signaling results in increased HIV expression in monocytes obtained from transgenic mouse spleen cells [33]. However, in latently infected ACH-2 T cells, a TLR9 agonist, ODN2006, strongly activated HIV transcription, but inhibited productive HIV infection in MT4 T cells and primary T cells [34]. In a model of latently infected mature mast cells, stimulation of TLR2, 4 or 9 triggered HIV-1 replication [35]. Thibault et al. [36] reported that TLR5 stimulation is sufficient to trigger reactivation of latent HIV-1 provirus in Jurkat T cells and to also activate viral gene expression in central memory CD4^+^ T cells. Consistent with these results, Brichacek et al. [37] found that activation of TLR5, but not TLR9, triggers HIV-1 transcription in lymphoid tissue ex vivo. The differential effects of these TLR ligands on HIV-1 replication correlated with changes in production of CC and CXC chemokines in the ligand-treated HIV-1-infected tissues [37]. More recently, Novis et al. reported that Pam3CSK4 (TLR2/1 agonist) leads to viral reactivation from latency in cultured central memory T (T_CM_) cells, suggesting a unique pattern of TLR-mediated HIV reactivation in primary T cells [38]. It has been known that TLR3 specifically recognizes bacterial ribosomal RNA [39], but its role in HIV transcriptional regulation in known.

In this study we investigated the effects of a wide range of TLR ligands, including potent TLR2 agonist molecules purified from *Mycobacterium* tuberculosis (Mtb), on the reactivation of pro-viral HIV in a novel human model of latently-infected microglial cells. Surprisingly, we found that TLR3 ligation potently reactivated HIV in microglial cells, but not in monocytes or T cells, using an NF-κB-independent pathway that involves activation of the IRF3 transcription factor. Additionally, HIV was modestly reactivated in human microglial cells by engagement of TLRs 4, 5, and 6. More restricted reactivation patterns were observed after stimulation of TLRs 1 and 2, whereas no significant reactivation was obtained by activation of TLRs 7, 8, or 9. The unique responses of microglial cells to TLR ligands provide an important framework for understanding how circulating bacterial antigens and nucleic acids can enhance HIV replication in microglial cells and potentially exacerbate HAND.

## Results

### Characterization of hµglia/HIV (HC01) and (HC69) cells

The HIV latently-infected cells (hµglia/HIV) used in this study were derived from simian virus 40 large T antigen (SV40Tag)/human telomerase reverse transcriptase (hTERT)-immortalized human microglial cell lines prepared as described by Garcia-Mesa et al. [40]. These novel cell lines, which have characteristic microglial cell phenotypes, have superseded CHME-5 cells in our laboratory. CHME-5 cells were originally believed to be of human origin [41], and have been widely used as models for human microglial cells even until very recently [42, 43]. Unfortunately, our detailed molecular analyses of CHME-5 proved that they are actually rat cells, and were probably derived from a contaminating rat glioma [40].

**Fig. 1:**
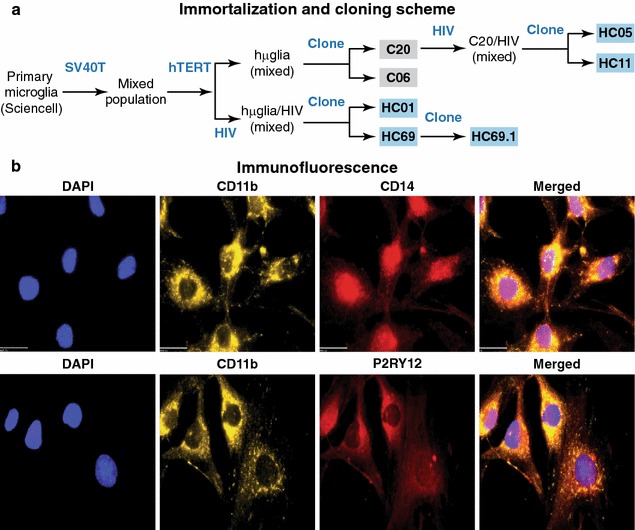
Isolation and characterization of hµglia/HIV (HC69) cells. **a** Schematic representation of a typical procedure to develop a microglia/HIV clonal cell population such as hµglia/HIV (HC69) cells. Uninfected clonal populations are indicated in *grey boxes*, and latently-infected clonal populations are indicated in *blue boxes*. **b** Immunofluorescence analysis of the human microglial cells hµglia/HIV (HC01). Cells were cultured, fixed, and immunostained with either anti-CD11b (*green*), anti-CD14 (*red*) or anti-P2RY12 (*red*) conjugated antibodies. Nuclei were stained with DAPI (*blue*). Merged images of nuclei, CD11b and CD14, or nuclei, CD11b and P2RY12 are indicated

**Fig. 2:**
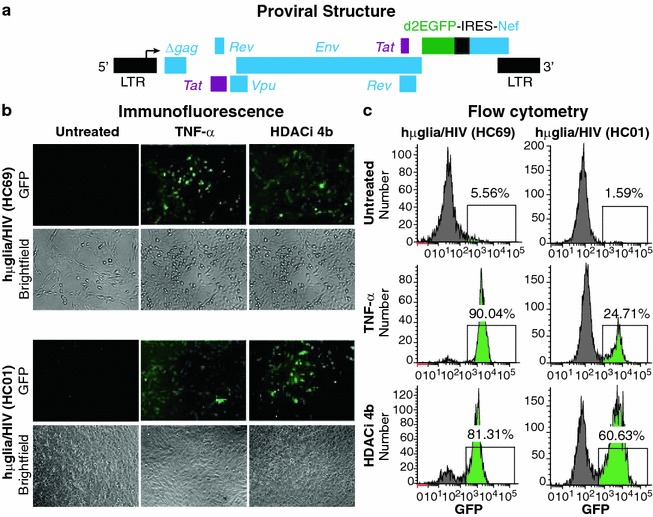
HIV emergence from latency in human microglial cell models. **a** Genome organization of the HIV lentiviral vector. A fragment of HIV-1_pNL4-3_, containing *Tat*, *Rev*, *Env*, *Vpu* and *Nef* with the reporting gene d2EGFP, is cloned into the pHR’ backbone. The resulted plasmid was used to produce the VSVG HIV particles, as described previously [112]. **b** Fluorescence microscopy analysis of TNF-α- and HDACi 4b-mediated reactivation of HIV in latently-infected microglial cells [hµglia/HIV (HC69) and (HC01)]. Cells treated with TNF-α (500 pg/mL) or HDACi 4b (30 µM). **c** FACS analysis 16 h post-treatment. In these, and subsequent FACS profiles, GFP^+^ cell populations are indicated in *green*, and the proportion of GFP-expressing cells is indicated in %

To prepare latently infected cells, we followed a strategy used successfully to create latently infected Jurkat T cell lines [44, 45] and CHME-5 cell lines [46]. Briefly, as depicted in Fig. 1a, and as extensively explained in Garcia-Mesa et al. [40], commercially-available primary microglial cells were immortalized through infection with vesicular stomatitis virus G (VSV-G) envelope pseudotyped viruses expressing SV40Tag and hTERT. Immortalized cells (hµglia) were either used to isolate clonal populations, such as C20 and C06 [40] or infected with a VSV-G pseudotyped lentivirus vector (PHR1′/d2EGFP) expressing the short-lived green fluorescence protein (d2EGFP) as a reporter construct (Fig. 2a) to develop clonal populations of hµglia/HIV, such as HC01 and HC69. In general, cells expressing GFP then were selected by cell sorting. In approximately 3–4 weeks, latently infected (GFP^−^) cells begin to outgrow the GFP^+^ cell population. The two clones hµglia/HIV (HC69) and hµglia/HIV (HC01), carrying latent proviruses, were then isolated from the GFP^−^ population.

The proviral integration site for each of these two clones has been sequenced and located within the host genome (Table 1). Both HC01 and HC69 are single integrants and, as expected from the extensive studies characterizing HIV proviral integration sites [44, 47–49], the provirus was located in the introns of host genes. The uninfected cell line C20 was used as a negative control, where no HIV sequence was detected.

**Table 1:** Proviral integration sites

Cell Type	Clone	LTR	Tat	Number of intergrants	Loci	Gene name	Intron or exon
Microglia	HC01	WT	H13L	1	chr2:95961496–95961523	ANKRD36C	Intron
Microglia	HC69	WT	H13L	1	chr9:7770778 6–77707849	nGNAQ	Intron

As shown in Fig. 1b, hµglia/HIV cells, exemplified by the clone HC01, express the well-established microglial surface markers CD11b and P2RY12 [50]. The cells also express the macrophage lineage marker CD14, suggesting that they display an activated phenotype, which is consistent with RNA-seq analyses of the hµglia (C20) cells [40].

### Induction of HIV expression in hµglia/HIV cells

The presence of latent HIV proviruses (Fig. 2a) in individual hµglia/HIV clones was confirmed by viral reactivation in the overnight presence of 500 pg/mL of tissue necrosis factor α (TNF-α) or 30 µM of the histone deacetylase inhibitor 4b (HDACi 4b) [51]. Induction of GFP was monitored by immunofluorescence (Fig. 2b) as well as flow cytometry results (Fig. 2c). In these cells, basal HIV expression was extremely low, with 1–6% of the cells expressing GFP. However, after exposure to TNF-α for 16 h, HIV was induced in approximately 90% of the HC69 cells, or 25% of the HC01 cells. Similarly, exposure to HDACi 4b resulted in a strong induction of HIV in nearly 81% of the HC69 cells, or 61% of the HC01 cells. In general, HC01 cells displayed a somewhat more restricted HIV reactivation profile than HC69 cells (Fig. 2b, c).

Parallel control experiments in monocytic cell lines used latently infected THP-1, U937, and SC cells. As shown in Additional file 1: Fig. S1, representative clones derived from each of these parental monocytic cell lines THP-1/HIV (HA3), U937/HIV (HUC5), and SC/HIV (HSCC4) cells, as well as Jurkat/HIV 2D10 [44], were highly responsive to treatment with 10 ng/mL TNF-α, with more than 95% of the HA3, HUC5 and 2D10 cells induced to express GFP. The HSCC4 cells were more restricted with approximately 50% of the cells expressing GFP after TNF-α treatment. There were a wide range of responses of these cells to LPS: THP-1/HIV (HA3) cells (96%) > U937/HIV (HUC5) (53%) > SC/HIV (HSCC4) (22%) > Jurkat/HIV 2D10 (3.5%). As expected, only the Jurkat T cells were activated through the TCR (α-CD3/α-CD28 mAb).

### TLR-mediated HIV reactivation of latently-infected human microglial

Microglial cells are known to express all TLRs at low, but detectable, levels [22]. Because engagement of TLRs can lead to NF-κB pathway activation, and in turn, HIV expression, we assumed that treatment of latently-infected microglial cells with selective TLR agonists would lead to viral reactivation provided there was sufficient receptor expression. However, as described below, microglial cells show specific restrictions in their responses to TLR agonists.

**Fig. 3:**
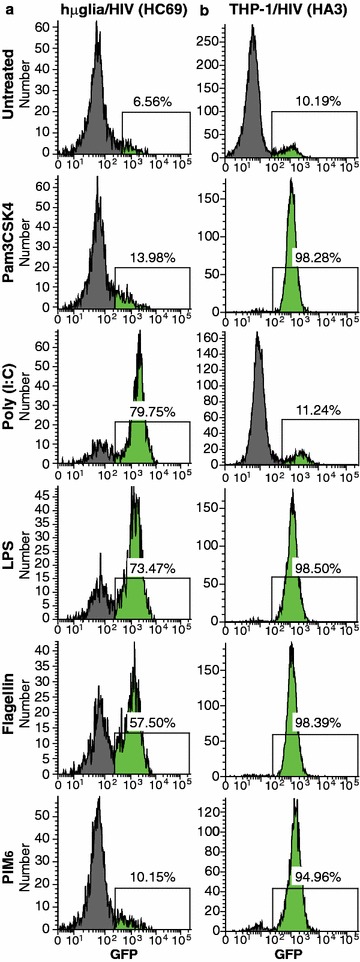
HIV reactivation by TLR agonists in latently-infected microglial cells. Treatment of the hµglia/HIV (HC69) clonal populations with TLR ligands. HC69 cells (**a**) were plated 8 h before no treatment or treatment with the TLR agonists Pam3CSK4 (1 µg/mL), poly (I:C) (10 µg/mL), LPS (5 µg/mL), flagellin (5 µg/mL) or PIM_6_ (5 µg/mL) for 16 h prior to measuring GFP expression by FACS analysis. THP-1/HIV (HA3) cells (**b**) were used as positive control

Efficient HIV reactivation in the permissive clone HC69 was achieved by LPS in up to ~73%, by flagellin in up to ~57% (Figs. 3a, 5a), and FSL-1 in up to ~52% (Fig. 5a). In the more restrictive clone HC01, HIV reactivation was achieved in the presence of LPS (~17%), flagellin (~13%) (Fig. 5a, and Additional file 2: Fig. S2a), and FSL-1 (~21%) (Fig. 5a). Pam3CSK4 (~14% in HC69; Fig. 3a, and ~8% in HC01; Additional file 2: Fig. S2a) and HKLM (~10% in both HC69 and HC01; Fig. 5a) only weakly reactivated HIV.

Surprisingly, poly (I:C) very potently reactivated HIV in hµglia/HIV (HC69) cells (~80%; Fig. 3a) and significantly in hµglia/HIV (HC01) cells (~21%; Additional file 2: Fig. S2a). No reactivation was observed with ligands for the rest of the TLRs (Fig. 5a).

In comparison, in rat hT-CHME-5/HIV (HC03) cells, poly (I:C) (~22%), LPS (~24%), and flagellin (~41%) were moderate activators of HIV (Fig. 5a; Additional file 2: Fig. S2b). The profile of HIV reactivation by TLR ligands in hT-CHME-5 (HC14) cells was similar to that of hT-CHME-5 (HC03) cells, with the exception of poly (I:C), which did not reactivated HIV in the HC14 cells (Fig. 5a). Weak or no reactivation was observed with the rest of the agonists in the rat cells, exemplified here with hT-CHME-5 (HC14) (Fig. 5a). In both the human and the rat cells, Mtb-derived TLR agonists were ineffective or very weak activators of HIV transcription (Additional file 3: Fig. S3a).

**Fig. 4:**
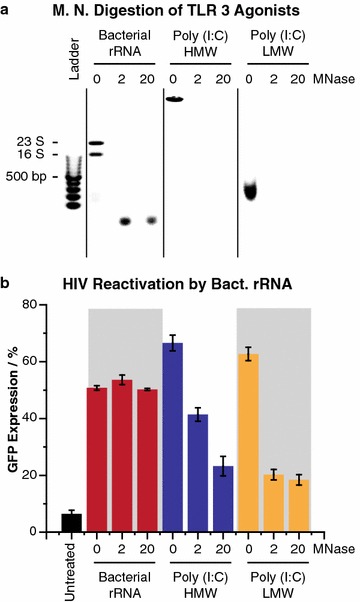
Effect of bacterial rRNA on HIV reactivation in microglia. **a** Microccocal nuclease (MNase) digestion of TLR3 agonists. Bacterial rRNA, poly (I:C) HWM, and poly (I:C) LMW were digested with 2 or 20 U of MNase. Undigested RNA and the digestion products were run on a 0.7% agarose gel. **b** HIV expression in HC69 cells by TLR3 agonist. Cells were incubated overnight with rRNA, poly (I:C) HMW, and poly (I:C) LMW undigested or digested with indicated doses of MNase. *Error bars* indicate the standard deviation for three or more experiments

**Fig. 5:**
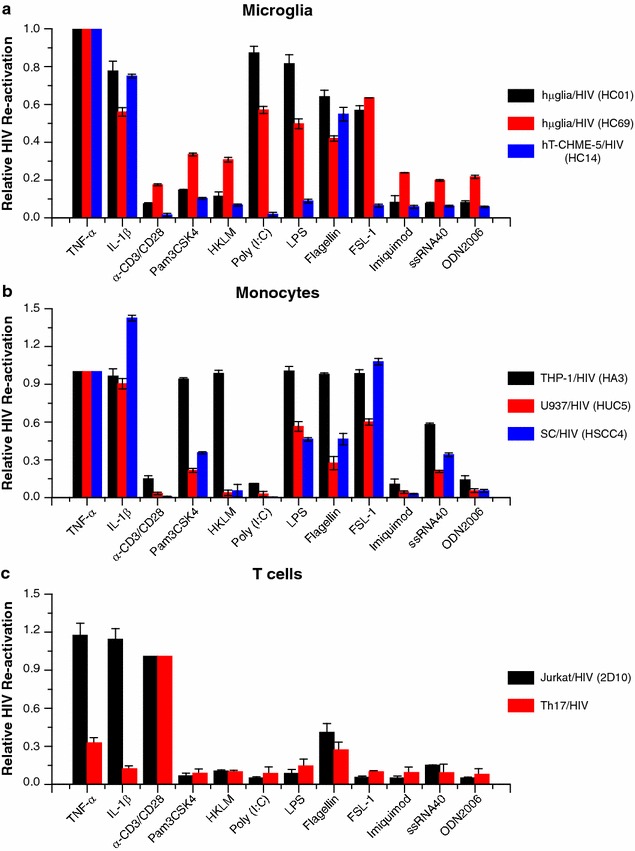
Relative induction (Y-axis) of HIV transcription by TLR ligands (X-axis). **a** Microglial cells are represented by hµglia/HIV (HC01; *black bars*), hµglia/HIV (HC69; *red bars*), and hT-CHME-5/HIV (HC14; *blue bars*). To compare the different cell lines, the data was normalized to TNF-α induction (100%). **b** The monocytic cells are represented by THP-1/HIV (HA3; *black bars*), U937/HIV (HUC5; *red bars*), and SC/HIV (HSCC4; *blue bars*). To compare the different cell lines, the data was normalized to TNF-α induction (100%). **c** T cells are represented by Jurkat/HIV (2D10; *black bars*) and Th17/HIV (mixed population; *red bars*). The data was normalized to α-CD3/CD28 induction levels (100%). *Error bars* indicate the standard deviation for three or more experiments

As a positive control, we also tested the ability of TLR agonists to induce HIV induction in the monocytic cell lines THP-1/HIV (HA3) (Figs. 3b, 4b), U937/HIV (HUC5) and SC/HIV cells (HSCC4) (Fig. 4b). In contrast to the microglial cells, the monocytic cells were unresponsive to poly (I:C) (TLR3 ligand), and both cell types were unresponsive to imiquimod (TLR7 ligand) or ODN2006 (TLR9 ligand) (Figs. 3b, 5b). Also, ssRNA40 (TLR8 ligand) was a weaker activator in microglial cells than in monocytes, and HKLM (TLR2 agonist) was only effective in THP-1/HIV (HA3) cells and, to a lesser extent, in hµglia/HIV (HC69) cells (Fig. 5a, b). In T cells, exemplified here by Jurkat/HIV (2D10) and Th17/HIV, only flagellin (TLR5 agonist) significantly reactivated HIV (Fig. 5c).

### Microglial cells respond poorly to potent TLR2 agonists derived from Mtb

Because of the poor responses of microglial cells to classical TLR2/1 ligands, we decided to also test a set of highly potent TLR2 agonists derived from Mtb. Mtb-derived lipoproteins such as LprG [52] as well as the glycolipid lipomannan (LM; [53–55]), but not mannosylated lipoarabinomannan (ManLAM; [53]), are potent TLR2 agonists that trigger pro-inflammatory and microbicidal innate immune responses. In addition, the Mtb-derived polar glycolipid phosphatidylinositol mannoside (PIM) 6 (PIM_6_), but not PIM_1,2_, triggers TLR2 signaling [56]. PIM_6_ (Fig. 3; Additional file 3: Fig. S3) or the other Mtb glycolipids or LprG tested did not reactivate HIV in microglial cells (HC01, HC69, and HC14) (Additional file 3: Fig. S3a).

In contrast to microglial cells, HIV was strongly reactivated by PIM_6_ or LprG in THP-1/HIV (HA3) cells (Additional file 3: Fig. S3b) in a dose-dependent manner (Additional file 5: Fig. S5). PIM_1,2_, LM and ManLAM were weak activators of HIV in THP-1/HIV (HA3) cells whereas not activators (Additional file 3: Fig. S3). Unexpectedly, LM failed to reactivate HIV in THP-1/HIV (HA3) cells (Additional file 3: Fig. S3). PIM_6_ and LprG were unable to reactivate HIV in U937/HIV (HUC5) or SC/HIV (HSCC4) cells as strongly as in THP-1/HIV (HA3) (Additional file 3: Fig. S3), presumably due to the limited levels of TLR2 receptor in the former (Additional file 4: Fig. S4).

### Bacterial rRNA, like poly (I:C) agonists, reactivate HIV in hμglia/HIV cells

It is well established that TLR3 is able to response to dsRNA, particularly dsRNA of viral origin. The effect on TLR3 activation by rRNA from bacteria is less documented, although some evidence indicates that dsRNA isolated from bacterial cultures engages TLR3 signaling [39]. Here, we show that rRNA purified from bacteria (Fig. 4a) reactivated HIV in HC69 cells up to ~50%, which is comparable to the reactivation obtained with poly (I:C) (both LMW and HMW; ~60–65%) (Fig. 4b). MNase digestion of bacterial rRNA at these concentrations resulted in accumulation of small dsRNA fragments (Fig. 4a), which still reactivate HIV. By contrast the poly (I:C) was completely digested (Fig. 4a) and could not reactive HIV (Fig. 4b).

### TLR expression on microglial/HIV and monocytic cells does not correlate with HIV reactivation

As described above, the HIV-latently infected monocytic cell lines responded more robustly to TLR stimulation than microglial cells, with the exception of TLR3 activation by poly (I:C) (Figs. 3, 5; Additional file 3: Fig. S3). Surprisingly, this did not strictly correlate with TLR expression patterns.

**Fig. 6:**
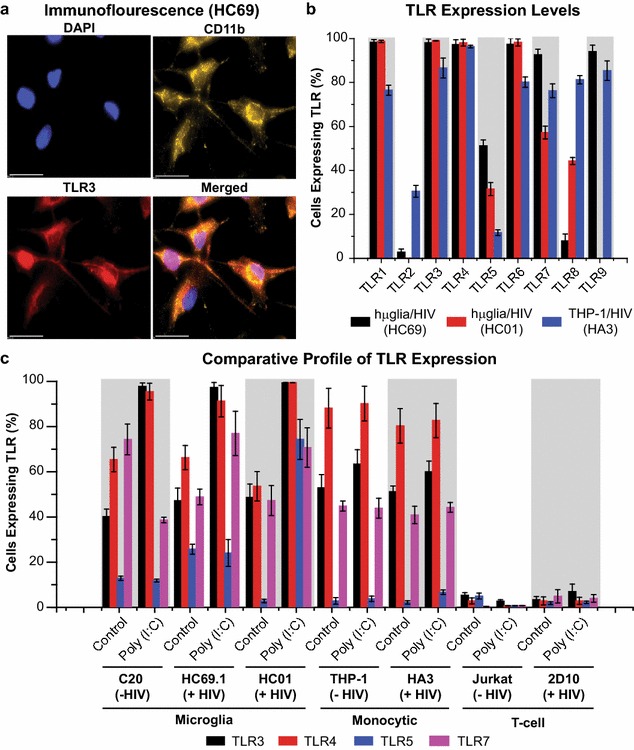
TLR expression on hµglia/HIV (HC69) and (HC01) cells. **a** Expression of TLR3 on clone HC69 by immunofluorescence. Cells were cultured, fixed, and immunostained with either anti-CD11b (*green*) or anti-TLR3 (*red*) conjugated antibodies. Nuclei were stained with DAPI (*blue*). Merged images of nuclei, CD11b and TLR3 are indicated. **b** Flow cytometry analysis quantification of surface expression of TLR1–9 on hµglia/HIV (HC69; *black bars*) and (HC01; *red bars*) cells. Cells were incubated with antibodies against TLR1–9, or corresponding isotype control. THP-1/HIV (HA3) cells (*blue bars*) were used as control. **c** Surface expression of TLR3, 4, 5, and 7 on indicated cell lines. Serum-starved cells were untreated or treated with 100 ng/mL of poly (I:C) prior to incubation with antibodies against these TLRs, or corresponding isotype control. *Error bars* represent the standard deviation of three or more experiments

Because poly (I:C) resulted in an unexpectedly strong activation of HIV in latently-infected microglial cells, we confirmed that TLR3 is expressed in both clone HC69 (Fig. 6a, b) and clone HC01 (Fig. 5b). Although almost 90% of the THP-1/HIV (HA3) cells expressed TLR3 (Fig. 5b), they failed to respond to poly (I:C) (Figs. 3b, 5b).

The level of expression of the rest of the individual TLRs (TLR1, 2, 4–9) on hµglia/HIV (HC69) and (HC01) cells, under normal growth conditions, was measured by flow cytometry, using THP-1/HIV (HA3) cells as a reference (Fig. 6b). Expression of TLR1 was significantly greater on hµglia/HIV (~99%, black and red bars) than on THP-1/HIV cells (~76%, blue bar); however, strong HIV reactivation by Pam3SCK4 is seen in the monocytes, but not in the microglia (Figs. 3, 5). This may be the result of the lack of TLR2 expression in microglia (~0.7–4%) compared to monocytes (~31%). Approximately 31% of THP-1/HIV (HA3) cells were positive for TLR2 expression, but there was no significant TLR2 expression in either HUC5 or HSCC4 cells (Additional file 4: Fig. S4), explaining the lack of PIM_6_- or LprG-mediated HIV reactivation (Additional file 3: Fig. S3). The lack of TLR2 expression is also consistent with the weak responses provoked by FSL-1 (TLR2/6 agonist; Fig. 5a, b) on HIV reactivation in hµglia/HIV versus monocytes/HIV cells, since TLR6 was actually expressed at a higher level (~99%) in the microglia than in the monocytes (~82%) (Fig. 6b).

Despite the relatively weak responses to LPS (Figs. 3a, 5a), almost the entire microglial cell population was positive for TLR4 expression (Fig. 6b). One important observation when comparing the LPS-responses of the three monocytic cell models was that the THP-1 response was stronger than U937, which in turn was stronger than SC. THP-1 cells were ~98% positive for TLR4 (~96% reactivated HIV by LPS), whereas only ~62% of U937 cells (~53% reactivated HIV by LPS) and ~25% of SC cells (~22% reactivated HIV by LPS) expressed the receptor (Additional file 4: Fig. S4), paralleling the hierarchy of responses of these cell types to LPS (Fig. 5b).

hµglia/HIV cells are positive for CD14, as exemplified by the HC01 staining (Fig. 1b). CD14 is the TLR4 co-receptor needed for LPS-mediated activation, ruling out the possibility that the relatively weak TLR4-mediated HIV transcriptional response was due to the absence of CD14. Therefore, the expression profiles of TLR4 on hµglia/HIV cells did not correlate with the more restricted HIV reactivation induced by LPS compared to monocytes.

A similar situation exists for the TLR5 receptor. A greater proportion of the microglial line expressed TLR5 (~32–53%) compared to the monocytic line (~12%; Fig. 6b), but the monocytes, nonetheless, responded more robustly to flagellin than the microglial cells.

Both cell types also expressed relatively high levels of TLR7 (Fig. 6b), but neither cell type responded to imiquimod (Fig. 5a, b). On the other hand, the high level of TLR8 expression on the monocytes (~82%) relative to the microglia (~9–45%) (Fig. 6b) can explain the higher response of HIV reactivation on monocytes by ssRNA40 (Fig. 5b). Finally, the differential TLR9 expression observed between the two microglial cell lines (~1 vs. ~95%), and compared with the monocytes (~87%) (Fig. 6b), does not explain that ODN2009 was not able to reactivate HIV in either of these cell lines (Fig. 5a, b).

### Modulation of TLR expression by poly (I:C)

In order to assess the ability to poly (I:C) to modulate TLR expression in the context of HIV infection, and potentially influence cellular responses to inflammation, we measured the effect of poly (I:C) treatment on the expression of TLR3, 4, 5, and 7 on uninfected C20 cells, and compared it to that on infected cells (HC69.1 and HC01) as well as monocytic and T cells (Jurkat) without and with HIV. Cells were serum-starved to reduce the overall levels of TLR expression, since they were at maximal levels under normal growing conditions (1–5% FBS). Poly (I:C) treatment increased the expression of TLR3 (from ~40 to 98%) and TLR4 (from ~65 to 97%), decreased the expression of TLR7 (from ~70 to 40%), and did not affect the expression of TLR5 (Fig. 6c).

In comparison with uninfected microglia (C20), cells latently infected with HIV (HC69.1 and HC01), showed only small differences in the basal levels of TLR expression for TLR3, 4, 5 and 7 (Fig. 6c). However, when the infected cells were stimulated with poly (I:C), the level of TLR3 (HC69.1: from ~50 to 98%; HC01: from ~50 to 99%), TLR4 (HC69.1: from ~70 to 90%; HC01: from ~55 to 99%), and TLR7 (HC69.1: from ~50 to 75%; HC01: from ~50 to 70%) increased. The level of TLR5 only increased in HC01 cells (from ~5 to 75%), but not in HC69.1 (remained at ~25%) (Fig. 6c).

A similar analysis was performed in monocytic cells (THP-1) with and without HIV in either the presence or the absence of poly (I:C) (Fig. 6c). Here, poly (I:C) treatment of uninfected cells (THP-1) increased the expression of TLR3 (from ~50 to 60%), but not of the other three receptors. In comparison with uninfected monocytic cells, cells latently infected with HIV (HA3) displayed similar TLR expression profiles (Fig. 6c). When the infected cells were stimulated with poly (I:C), the level of all four receptors only slightly increased: TLR3 from ~50 to 60%, TLR4 from ~80 to 83%, TLR5 from ~5 to 10%, and TLR7 from ~40 to 45% (Fig. 6c). In T cells, using Jurkat as a model, all four receptors were only slightly altered in infected cells (2D10), and poly (I:C) seemed to play a minor to an insignificant role in modulating the expression of any of these four receptors.

### Transient induction of NF-κB by TLR ligands in microglial cells

**Fig. 7:**
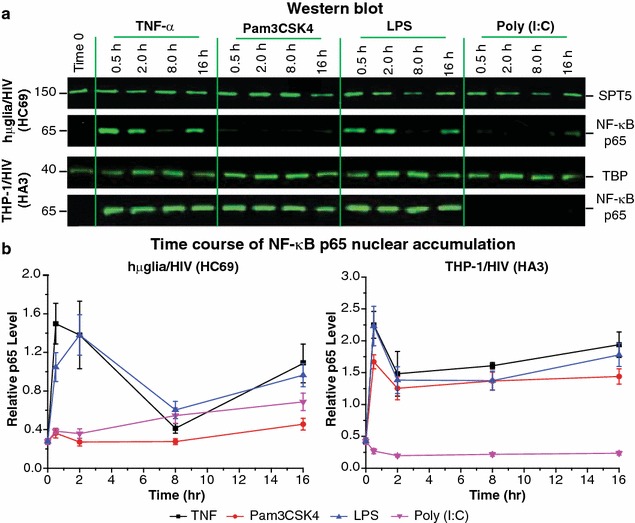
Induction of NF-κB nuclear recruitment by TLR ligands in hµglia/HIV (HC69) cells. **a** Representative Western blot analysis images of NF-κB p65 nuclear recruitment after stimulation. Cells [hµglia/HIV (HC69), and THP-1/HIV (HA3), as control] were untreated or treated with TNF-α (10 ng/mL), Pam3CSK4 (1 µg/mL), LPS (1 µg/mL) or poly (I:C) (1 µg/mL) for 30 min, 2, 8, or 16 h prior to nuclear extracts (NE) purification. Anti-SPT5 antibody for hµglia/HIV and anti-TBP antibody for THP-1/HIV were used as loading control. Molecular weights are indicated at the *left* of the blots. **b** Quantification of NF-κB p65 nuclear recruitment is depicted in the relative p65 band intensity (Y-axis) versus time (X-axis) graphs. TNF-α is shown in *black squares*, Pam3CSK4 in *red circles*, LPS in *blue triangles*, and poly (I:C) in *purple triangles*. *Error bar* represents the standard deviation of three or more experiments

The HIV LTR carries two tandem NF-κB binding motifs [57], which are required for proviral emergence from latency in T cells [27, 29], but are dispensable for viral growth in most T cell lines [26]. One possible explanation for the inefficient responses of microglial cells to TLR ligands is that induction of the NF-κB p65 subunit nuclear translocation is inefficient or short-lived. Nuclear NF-κB p65 accumulation was therefore measured by Western blot analysis following TLR activation. hµglia/HIV (HC69) and THP-1/HIV (HA3) cells were treated with TNF-α (10 ng/mL; positive control), Pam3CSK4 (1 µg/mL; TLR2/1), LPS (1 µg/mL; TLR4), or poly (I:C) (1 µg/mL; TLR3) and monitored at the 30 min, 2 h, 8 h, or 16 h time points. The results (Fig. 7) demonstrated that, while TNF-α (Fig. 7b, black square/line) and, to a lesser extent LPS (blue triangle/line), strongly induced p65 nuclear translocation in hµglia/HIV cells at the 0.5 and 2-h time points and again at the later 16-h time point, Pam3CSK4 (red circle/line) and poly (I:C) (purple triangle/line) only weakly induced p65 nuclear translocation at the 16-h time point. In contrast, in THP-1/HIV cells treated with TNF-α, Pam3CSK4, or LPS, p65 strongly appeared at the 30-min time point and remained in the nucleus until the 16-h time point. In THP-1/HIV cells, poly (I:C) also failed to recruit p65 (Fig. 7a, b).

To confirm the requirement for NF-κB in TLR-mediated HIV reactivation in latently-infected microglial cells, CHME-5 and THP-1 cells were infected with a PHR’ derivative carrying inactivating mutations in the NF-κB binding site of the HIV LTR [29] (CHME-5/HIV_mNF-κB or THP-1/HIV_mNF-κB). As shown by flow cytometry analysis (Additional file 5: Fig. S5), flagellin was unable to reactivate HIV in either CHME-5/HIV_mNF-κB or THP-1/HIV_mNF-κB cells, as compared to the potent activation achieved in CHME-5/HIV (H1F3) or THP-1/HIV (HA3) cells. By contrast, HDACi 4b was a potent inducer of the proviruses in both the cells carrying the wild type virus and the cells carrying the mutant virus (Additional file 5: Fig. S5).

In complementary control experiments, the reporter THP1-XBlue™ cells (Invitrogen), which carry an embryonic alkaline phosphatase under the control of a synthetic NF-κB-inducible promoter, were used to measure NF-κB induction by TLR agonists. The results (Additional file 6: Fig. S6a) confirmed that only the TLR agonists that reactivated HIV in THP-1/HIV (HA3) cells induced NF-κB activation in a time-dependent manner in THP1-XBlue reporter cells; this reactivation was blocked in the presence of DRB and flavopiridol, inhibitors of P-TEFb (Additional file 6: Fig. S6b; please, read further below). Consistently, Western blot analysis of nuclear p65 levels in THP-1/HIV (HA3) cells treated for 30 min with TNF-α or indicated TLR agonists demonstrated that the TLR agonists that reactivated HIV were those that were able to induce NF-κB nuclear recruitment (Additional file 6: Fig. S6c).

Similarly, in THP-1/HIV (HA3) cells, HIV reactivation by TLR ligands was effectively blocked using the IKKγ NEMO binding domain (NBD) inhibitory peptide, but not the control peptide (Imgenex, CA) (Additional file 7: Fig. S7a).

### Induction of IRF3 in microglial cells by TLR3 ligands

**Fig. 8:**
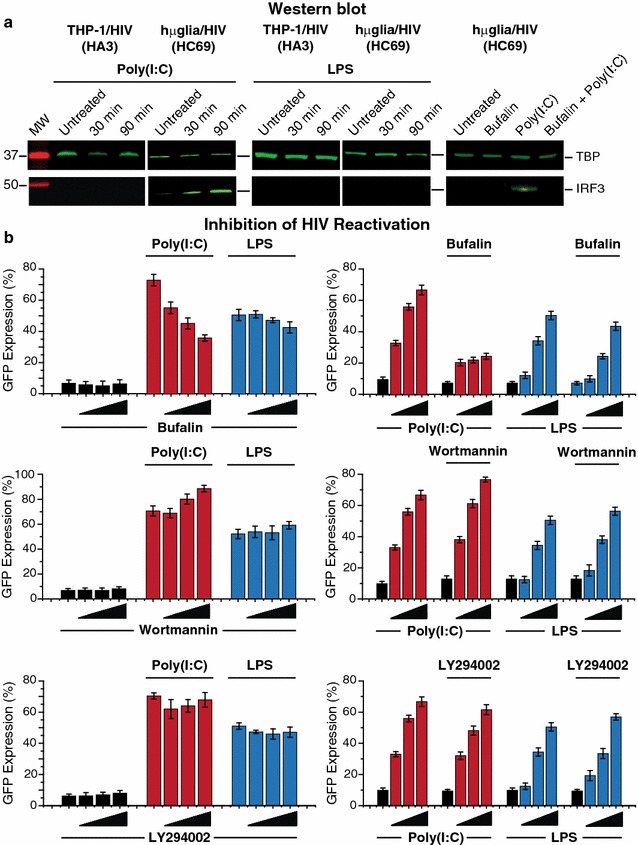
Poly (I:C)-mediated HIV reactivation in hµglia/HIV (HC69) cells requires IRF3 nuclear recruitment. **a** Representative Western blot analysis images of IRF3 nuclear recruitment after poly (I:C) stimulation. Cells were untreated or treated with poly (I:C) (1 µg/mL), or LPS (1 µg/mL), as negative control, for 30 or 90 min prior to nuclear extracts purification. *Far right* Representative Western blot analysis images of IRF3 nuclear recruitment after poly (I:C) stimulation in the absence or presence of bufalin. Cells were untreated or treated with poly (I:C) (1 µg/mL), bufalin (25 nM), or a combination of both for 90 min prior to nuclear extracts purification. For all blots, anti-TBP antibody was used as loading control. Molecular weights of IRF3 and TBP are indicated at *left*. **b** Pharmacological inhibition of poly (I:C)-mediated HIV reactivation. *Left* hµglia/HIV (HC69) cells were untreated or pre-treated with either poly (I:C) (1 µg/mL) or LPS (500 pg/mL) for 30 min prior to addition of inhibitors [bufalin (0, 5, 10, and 25 nM); wortmannin (0, 0.5, 2, and 5 nM); LY294002 (0, 0.5, 2, and 5 µM)]. *Right* hµglia/HIV (HC69) cells were untreated or pre-treated with inhibitors [bufalin (25 nM); wortmannin (5 µM); LY294002 (5 µM)] for 30 min prior to no-addition or addition of either poly (I:C) (0, 0.1, 0.5, and 1 µg/mL) or LPS (0, 20, 100, and 500 pg/mL), as indicated

Surprisingly poly (I:C), which reactivated HIV in hµglia/HIV, but not in THP-1/HIV cells, did not induce p65 nuclear translocation in any of these cell lines (Fig. 7), indicating that an NF-κB-independent pathway is involved in TLR3-mediated HIV reactivation in microglial cells. We therefore tested whether poly (I:C) was capable of inducing IRF3, which could potentially trigger HIV reactivation either directly or indirectly. As shown in Fig. 8a, poly (I:C) induced IRF3 nuclear recruitment in hµglia/HIV (HC69) cells, but not in THP-1/HIV (HA3) cells. In contrast, LPS did not induce IRF3 nuclear translocation in either hµglia/HIV (HC69) or THP-1/HIV (HA3) cells (Fig. 8a).

To confirm the involvement of IRF3 in HIV induction in hµglia/HIV cells, we performed studies with the IRF3 modulator bufalin. Bufalin is a cardiotonic steroid that has been found to potently prevent IRF3 dimerization and nuclear localization [58]. We also tested the PI3-kinase inhibitors LY294002 and wortmannin, since both of these drugs can have indirect effects on IRF3 signaling. For example, LY294002 was found to inhibit LPS- and poly (I:C)-mediated IFN-β transcription and secretion, as well as IRF3 transcriptional activation and binding to the IFN-β promoter in fibroblasts [59]. By contrast, wortmannin did not inhibit IFN-β production in these studies [59]. Treatment of monocyte-derived dendritic cells (DC) with wortmannin or LY294002 enhanced IFN-β expression upon TLR3 or TLR4 engagement [60]. In the same study, it was reported that wortmannin-treated DC cells exhibited enhanced levels of IKK-α/β phosphorylation and IκB-α degradation with a concomitant increase in NF-κB nuclear translocation, as well as enhanced NF-κB activity induced by TRIF overexpression in HEK 293T cells [60].

As shown in Fig. 8a (right), the nuclear recruitment of IRF3 induced by poly (I:C) was blocked by bufalin. Bufalin also progressively inhibited HIV reactivation in response to poly (I:C) in HC69 cells (Fig. 8b). This effect can be seen when either the drug titrated and poly (I:C) and LPS are used at fixed concentrations (Fig. 8b, left-hand graphs) or when the inducers are titrated and bufalin was used at a concentration of 25 nM (Fig. 8b, right-hand graphs). As expected, there was no significant inhibition of LPS-mediated viral reactivation by bufalin (Fig. 8b). Treatment of the cells with the negative control, wortmannin, slightly enhanced responses to poly (I:C), but had no effect on LPS-mediated HIV reactivation (Fig. 8b). Similarly, LY294002 did not significantly inhibit or enhance HIV reactivation after poly (I:C) or LPS treatment (Fig. 8b).

### IRF3 is recruited to the HIV promoter upon activation with poly (I:C)

**Fig. 9:**
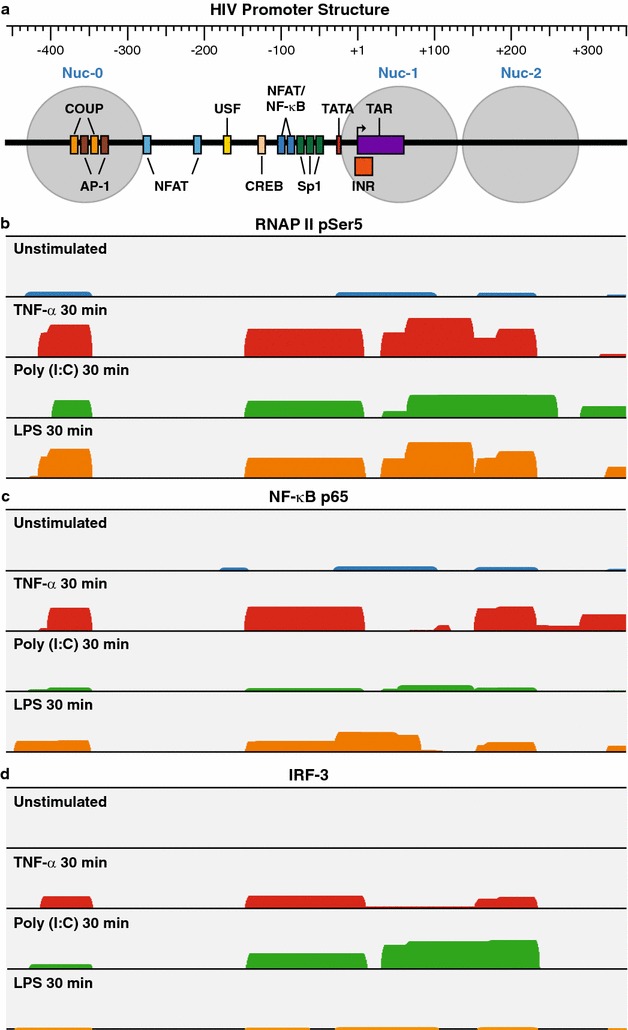
Chromatin immunoprecipitation assays showing the association of RNAP II (pSer5), NF-kB p65 and IRF3 with the HIV LTR. HC69 cells were untreated or treated with TNF-α (10 ng/mL), poly (I:C) (100 ng/mL) or LPS (10 ng/mL) for 30 min. DNA–protein complexes were extracted from formaldehyde-crosslinked cells. **a** Schematic representation of the HIV promoter region. **b** Histograms of sequence reads mapping to the HIV LTR representing the distribution and relative abundance of RNAP II pSer5, **c** p65, **d** IRF3

In light of the involvement of IRF3 in regulating poly (I:C)-mediated HIV reactivation in microglial cells, we performed chromatin-immunoprecipitation (ChIP) analysis on HC69 cells untreated or treated with either TNF-α (positive control), poly (I:C) or LPS for 30 min (Fig. 9). To obtain objective information about the distribution of the transcription factors on the HIV proviral DNA we utilized a new ChIP-Seq protocol in which we selected for HIV sequences by hybridization prior to sequencing. The reads were then mapped to the HIV LTR.

Isolated crosslinked DNA–protein samples were subjected to immunoprecipitation with anti-IRF3 and anti-NF-κB p65 antibodies. Anti-RNAP II pSer5 antibody was used as control. There was minimal RNAP II pSer5 detected on the HIV LTR in the latently infected cells. As expected, RNAP II (Fig. 9b) was recruited to HIV promoter in response to TNF-α, poly (I:C) or LPS. The RNAP II accumulated at the promoter and promoter proximal sites downstream of TAR, which mirrors the distribution patterns seen in induced T cells [61].

Upon treatment with poly (I:C) (Fig. 9d), IRF3 is recruited to the HIV promoter region (Fig. 9a). IRF3 accumulated preferentially in the −100 to +200 region, suggesting the presence of a binding site in this region. By contrast, NF-κB p65 (Fig. 9c) was practically absence at the HIV promoter in the presence of poly (I:C).

Treatment with LPS yielded opposite results: the abundance of NF-κB p65 at the HIV promoter, especially in the −100 to +1 region (Fig. 9c), which overlaps the two tandem NF-κB binding site in the HIV enhancer, was strongly increased after LPS treatment. There was also some accumulation NF-κB p65 in downstream regions, consistent with previous reports of transcription factor binding in the promoter proximal region in myeloid lineage cells [62, 63]. As expected from our Western blot analyses (Figs. 7, 8), LPS treatment did not lead to IRF-3 recruitment.

Treatment with TNF-α strongly induced NF-κB p65 recruitment (Fig. 9c) to the HIV promoter region but recruitment of IRF3 by TNF-α was considerably weaker than by poly (I:C) (Fig. 9d).

### P-TEFb components are constitutively expressed in microglial cells

Tat-dependent HIV expression is strictly dependent upon P-TEFb [27, 64]. Since CycT1, a subunit of P-TEFb, is known to be up-regulated during early monocyte differentiation, and then subsequently down-regulated in mature macrophages [65], we were concerned that poor responses to TLR agonists could be associated with limiting P-TEFb levels in microglial cells. There could also potentially be upregulation of CycT1 levels in response to TLR activation.

**Fig. 10:**
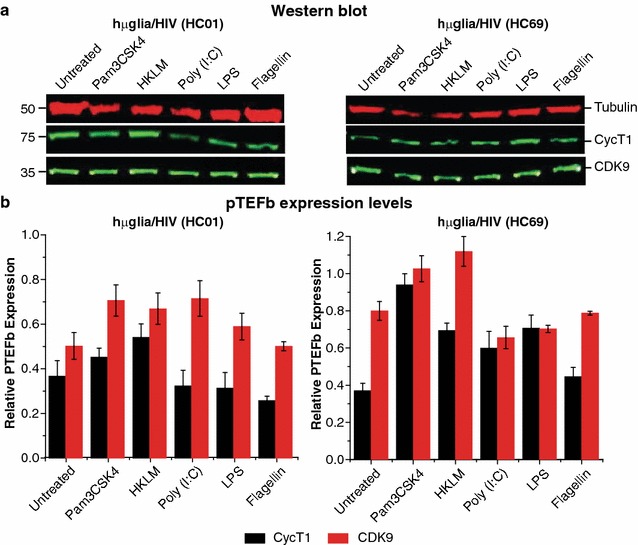
TLR-mediated activation and NF-κB nuclear translocation are not accompanied by significant P-TEFb production induction in hµglia/HIV cells. **a** Representative Western blot analysis blots of CycT1 and CDK9 expression in whole cell extracts (WCE) from hµglia/HIV (HC01) and (HC69) cells treated with TLR agonists. Cells were incubated with indicated TLR agonists (Pam3CSK4 at 1 µg/mL, HKLM at 10^8^ cells/mL, poly (I:C) at 10 µg/mL, and LPS and flagellin at 5 µg/mL) for 16 prior to WCE preparation and SDS-PAGE/Western blot analysis with anti-CycT1 antibody, anti-CDK9 antibody, or anti-Tubulin antibody as loading control. **b** Bar graph depicts the relative level of CycT1 (*black bars*) and CDK9 (*red bars*) expression in each clone, with *error bars* representing the standard deviation of three experiments

We measured CycT1 levels in hµglia/HIV HC69 and HC01 cells, before and after TLR activation. As a control, we measured CDK9 levels, the second P-TEFb subunit, whose expression is maintained at high levels during and after macrophage differentiation [65]. Western blot analysis of both cell lines (Fig. 10a) untreated or treated with TLR ligands [Pam3CSK4, HKLM, poly (I:C), LPS, and flagellin] demonstrated that both CycT1 and CDK9 were stably and constitutively expressed in these cells. In HC69 cells, a slight induction of CycT1 was observed (Fig. 10b), especially with Pam3CSK4 (from ~0.4 to 1 arbitrary units), but in general, not significant augmentation of P-TEFb by the tested TLR agonists was detected. As expected, HIV reactivation in hµglia/HIV (HC01) and (HC69) and hT-CHME-5/HIV (HC03) and (HC14) cells was impaired by the P-TEFb inhibitors DRB or flavopiridol (Additional file 7: Fig. S7b).

### Sensitization of hµglia/HIV (HC01) cells by HDACi 4b, but not by pro-inflammatory stimuli, for TLR-mediated HIV reactivation

**Fig. 11:**
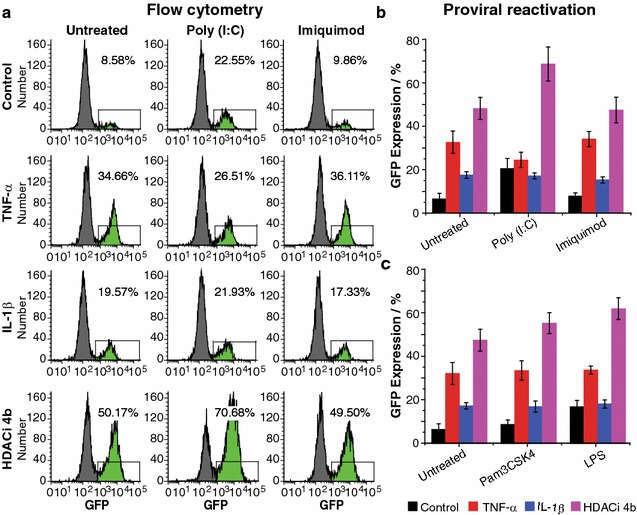
Effect of TLR agonists in combination with pro-inflammatory stimuli in HIV reactivation in hµglia/HIV (HC01) cells. Flow cytometry analysis of the effect of poly (I:C) or imiquimod treatment in combination with TNF-α or IL-1β on HIV emergence from latency. **a** hµglia/HIV (HC01) cells were untreated or treated with TNF-α (500 pg/mL), IL-1β (100 pg/mL), or HDACi 4b (25 µM) alone or in combination with poly (I:C) (1 µg/mL) or imiquimod (1 µg/mL) for 16 h prior to measuring GFP-expressing cells by flow cytometry. Fraction of cells expressing HIV is indicated by %. **b** Quantification of three or more combinatorial experiments is shown. Control *black bars*, TNF-α *red bars*, IL-1β *blue bars*, and HDACi 4b *purple bars*. Pam3CSK4 at 1 µg/mL and LPS at 5 µg/mL. *Error bars* indicate standard deviation of three or more experiments

Neuronal dysfunction does not correlate with the number of HIV-infected cells or viral antigens in CNS [66, 67], but rather with elevated inflammatory cytokine levels. High levels of interleukin (IL)-1β and TNF-α are seen in the CNS of patients with HAND [68, 69]. Also, a central role for TNF-α, IL-6, and IL-1β in gp120-induced neuroinflammation has been demonstrated using a rat model [70], where intrathecal administration of gp120 induced the expression of these cytokines. In addition, IL-8 has been reported to be increased during brain injury and neuroinflammation [71], and in human brain-derived endothelial cells and astrocytes by Tat [10, 38] and gp120 [72]. In light of the central role of these interleukins in mediating neuroinflammatory responses, we measured the ability of IL-1β, IL-6, and IL-8 to induce HIV reactivation in microglial/HIV cells. We found that IL-1β (Fig. 11; Additional file 8: Fig. S8), but not IL-6 or IL-8 (Additional file 8: Fig. S8), induced HIV reactivation in hµglia/HIV (HC01) (~20%), hµglia/HIV (HC69) (~72%), CHME-5/HIV (H1F3) (~22%), hT-CHME-5/HIV (HC03) (~21%), and hT-CHME-5/HIV (HC14) (~38%) cells.

Because hµglia/HIV (HC01) cells showed a more restrictive phenotype than hµglia/HIV (HC69) cells (Fig. 2), we wanted to determine whether the combination of pro-inflammatory stimuli with TLR ligands could potentiate HIV reactivation. Surprisingly, none of the TLR ligands tested, poly (I:C) and imiquimod (Fig. 11a, b), or Pam3CSK4 and LPS (Fig. 11c) showed additive effects with TNF-α or IL-1β. However, additive effects were seen using HDACi 4b in combination with poly (I:C) and, to a lesser extent, LPS (Fig. 11). By contrast, the negative control, imiquimod, did not increase the responses of HDACi 4b-treated cells. This result suggests that chromatin repressive structures on the HIV promoter may limit TLR-mediated HIV transcription.

## Discussion

### HIV can establish latency in a wide variety of myeloid cell types

In order to understand HIV expression regulation in the context of pro-inflammatory conditions occurring in CNS, we have developed a number of ex vivo models of HIV latency in microglial cells and, for comparison, in monocytic cell lines. Taking advantage of lentiviral reporters, we developed these cellular models representing three different cell types: hµglia/HIV (HC69) and (HC01) (Fig. 5; Additional file 3: Fig. S3) (human microglia); CHME-5/HIV (H1F3) (Additional file 5: Fig. S5), hT-CHME-5/HIV (HC03) and (HC14) (Additional file 7: Fig. S7) (rat microglia); THP-1/HIV (HA3), U937/HIV (HUC5) and SC/HIV (HSCC4) (Fig. 5; Additional file 3: Fig. S3) (human monocytes); and Jurkat/HIV cells (2D10) and primary Th17/HIV (Fig. 5; Additional file 3: Fig. S3) (human T cells).

In previous studies of HIV latency in microglial cells, we employed the CHME-5 cells [46], which were originally believed to be of human origin [73]. Unfortunately, CHME-5 cells are actually rat cells [40]. To study latency in *bona fide* human microglial cell lines, we have developed microglial cell lines from human primary glial cultures using SV40Tag or SV40Tag/hTERT-immortalization (hµglia; Fig. 1a, and [40]). Subsequently, we used the hµglia cells to create HIV-latently infected microglial cells (hµglia/HIV). The hµglia cell lines demonstrate a microglial phenotype and display surface markers and functional properties of primary microglia [40]. In particular, even after infection with HIV, these cells continue to express CD11b and P2RY12 [50] (Fig. 1b).

One important observation is that these microglial cell lines showed high level expression of CD14, which should be low in resting microglia, suggesting that the cells have an activated phenotype [72]. We speculate that one unexpected consequence of culturing microglial ex vivo is that it induces and permits sustained expression of CD14 even in the absent of classical stimuli such as TNF-α or IL-1β.

### HIV transcriptional control in microglial cells

The HIV promoter is an auto-regulated promoter, which requires the Tat protein to stimulate efficient transcriptional elongation. As a result of this feedback mechanism, HIV silencing occurs whenever Tat availability falls below certain threshold levels needed to sustain transcription [27, 74–76]. The decline in Tat levels is typically the result of multiple complementary inhibitory mechanisms. First, in latently infected cells, the transcription initiation factor NF-κB (or in T cells, NFAT) is sequestered in the cytoplasm. Second, the LTR acquires heterochromatic structures that block the transcription start site [44, 77]. Typically, latent proviruses accumulate high levels of histone deacetylases and deactylated histones [78–80], and methylated histones [44, 81–83]. Finally, in quiescent T cells and monocytic precursors, but not in microglial cells, the essential Tat-associated transcription elongation factor, P-TEFb, is severely reduced because of degradation of CycT1 [65, 84, 85].

Induction of HIV transcription requires reversal of the chromatin restrictions, which is typically the result of NF-κB induction and binding to its recognition sites in the HIV LTR region. Chromatin restrictions appear to be particularly important for maintaining HIV latency in microglial cells [44, 77–83]. Broad spectrum inhibitors of histone deacetylases or histone methylase transferases are usually potent inducers of latent proviruses [86, 87]. We have shown here that HDACi 4b [51], which is a chemically novel histone deacetylase inhibitor, is capable of reactivating HIV in the absence of NF-κB induction and is one of the most potent activators of HIV transcription in the microglial cells that we have evaluated (Fig. 2; Additional file 3: Fig. S3 and Additional file 8: Fig. S8). In view of these molecular mechanisms, it is not surprising that TLRs, which are potent inducers of NF-κB and AP-1, are also able to activate HIV transcription in latently infected microglial cells.

### Unique mechanisms of TLR-mediated HIV reactivation in microglial cells

We tested the ability of agonists for TLR1–9 to reactivate HIV in microglia as well as in monocytic and in T cells. Surprisingly, only the agonist for TLR3, and to a lesser extent for TLR4, 5 and 6, reactivated significantly HIV in latently-infected microglial cells (Figs. 3, 4, 5; Additional file 2: Fig. S2; Additional file 3: Fig. S3). The agonists for TLR4, 5 and 6 induced NF-κB p65 nuclear translocation, but the effects were transient and therefore proviral reactivation was inefficient.

The engagement of TLR3 resulted in the strongest HIV reactivation in human microglial cells of any of the TLRs (Fig. 5). We confirmed that in microglial cells, poly (I:C) did not induce the nuclear translocation of p65 (Fig. 7), but rather uniquely activated the IRF3 transcription factor (Figs. 8, 9). HIV reactivation by poly (I:C), but not by LPS, was significantly inhibited by the IRF3 activation inhibitor bufalin [58] (Fig. 8b), which precluded poly (I:C)-mediated IRF3 nuclear recruitment. This is consistent with earlier reports that LPS did not induce IRF3 nuclear translocation [88]. This suggests that an NF-κB-independent pathway, utilizing IRF3 activation pathway [89], may be responsible for mediating HIV reactivation in the presence of poly (I:C). There have been no previous reports of IRF3 activation of latent HIV proviruses. Indeed, the only previous reports of IRF3 effects on HIV replication come from Suh et al. [90], who found that ligands for both TLR3 and TLR4 inhibited HIV replication in microglia in an IRF3-dependent manner due to the induction of host restriction factors. Another report by Sang et al. [91] also indicated that activation of TLR3 inhibited SIV infection and replication in macaque macrophages through induction of viral restriction factors. More detailed studies will be needed to distinguish between the effects of TLR3 activation on pre-integration events, subject to host restriction, and post-integration proviral reactivation events of the type we have investigated.

These results were confirmed using ChIP experiments (Fig. 9). Treatment with poly (I:C), but not with LPS (Fig. 9) led to the selective recruitment of IRF3, but not of NF-κB, at the HIV LTR. Transcriptional regulation by IRF3 of NF-κB responsive genes has been observed in other contexts. For example, Wang et al. [92] showed that the expression of the ZAP gene was directly regulated by IRF3 following virus infection or stimulation of cells with dsRNA or dsDNA, and that interaction with ZAP promoter was not dependent on NF-κB. Similarly, Freaney et al. conducted a comprehensive ChIP-seq study, which provided a detailed and quantitative genome-wide analysis of transcriptional regulation of the cellular antiviral response, and revealed extensive colocalization of IRF3 and NF-κB during virus infection [93].

TLR3 is activated by double-stranded RNA (dsRNA) [94], an intermediate formed by most viruses during their replication phase, and functions as a signal to activate inflammatory cells [95, 96]. For example, in vitro transcribed HIV gag mRNA complexed with lipofectin activates TLR3 [97].

In the context of HIV infections, it is important to note that TLR3 responses can also be triggered by bacterial and other dsRNAs [97–99]. High levels of circulating bacterial rRNA, LPS, and other bacterial antigens is a hallmark of the chronic immune activation seen in HIV-infected patients, due to long-term damage to the gut. Since LPS and dsRNA can reach the brain, it seems likely that they can act in unison to reactivate HIV in infected microglial cells and thereby induce neuronal damage.

The signaling pathways initiated by dsRNA/TLR3 differ between human cells of separate lineages, as well as between mouse and human. Lundberg et al. [100] showed that TLR3-induced mechanisms of human primary dendritic cells, macrophages, endothelial cells, and synovial fibroblasts, while expressing TLR3 at comparable levels, differ substantially. For example, poly (I:C) induced IP-10 secretion by all cell types, while dendritic cells and macrophages failed to produce TNF-α and IL-6 and, unexpectedly, TNF-α was secreted only by synovial fibroblasts. Interestingly, these findings were specific for human cells, and not for murine cells.

Microglial cells are extraordinarily sensitive to activation by the pro-inflammatory cytokines TNFα or IL-1β, and the presence of these cytokines in the CNS correlates strongly with the development of HAND [69]. At low concentrations, these cytokines are capable of eliciting strong HIV responses. Surprisingly, we found no additive effects, or even cross-talk, between TNF-α or IL-1β, and TLR ligands (Fig. 11). However, when we used the chromatin-modifying agent HDACi 4b in combination with Pam3CSK4, poly (I:C), or LPS, but not imiquimod, HIV reactivation was stronger than each of the elements individually (Fig. 9). This strongly implies that chromatin remodeling is required for potent TLR-mediated responses.

### Attenuation of TLR-mediated activation of latent HIV

The level of TLR-mediated HIV reactivation varies widely between the different myeloid and T cell types (Fig. 5). Even though microglial cells can express the entire spectrum of TLR receptors (Fig. 6; [22]), they are relatively inefficient for the reactivation of HIV compared to THP-1 cells. In most cases, the poor responses in microglial cells do not correlate with levels of receptor expression. For example, hT-hµglia/HIV (HC01) cells express very high levels of CD14 (Fig. 1) and TLR4 (Fig. 6), but respond poorly to LPS (Fig. 5). In hµglia/HIV cells, engagement of TLR4 is able to transiently induce NF-κB, as exemplified by clone HC69 (Fig. 7), suggesting that specific feedback mechanisms dampen down NF-κB responses. By contrast, in monocytic cells the NF-κB response is sustained and HIV reactivation is enhanced.

In microglial cells (and astrocytes), unlike in the rest of the cells of the immune system, activation of the NF-κB pathway not only leads to expression of pro-inflammatory genes, but also to the recruitment of repressor complexes to the promoter of pro-inflammatory genes to prevent the mounting of exacerbated inflammatory responses in the brain, which can damage adjacent neurons [101, 102]. We are currently investigating whether specific co-repressors are also used to attenuate HIV responses to NF-κB in microglial cells.

We have also examined the effect of TLR ligands on HIV reactivation in latently-infected T cells. Our results demonstrate that activation of TLR5 weakly, but significantly, induce HIV expression in latently-infected Jurkat/HIV (2D10) cells as well as in primary Th17/HIV (Fig. 5). This is consistent with previous observations by Thibault et al. [36], that TLR5 stimulation is a potent activator of latent HIV-1 provirus in Jurkat T cells, and also activates virus gene expression in T_CM_. Novis et al. also reported that the TLR2/1 agonist Pam3CSK4 leads to viral reactivation from latency in cultured T_CM_, but in contrast to our results using both Jurkat and primary Th17 cells, there is no significant activation of these cells by TLR4 or TLR5 agonists [103].

## Conclusions

HIV does not infect neurons, but it is frequently found in perivascular macrophages and microglia [104]. HIV encephalitis correlates with the number of activated brain mononuclear phagocytes (both perivascular macrophages and microglia), but not with the amount of virus or the number of infected cells [105, 106]. Therefore, the major role of microglial cells in HAND development appears to be neuroinflammatory and neurotoxic, which is greatly potentiated by viral proteins shed by viral CNS sanctuaries [107].

Our data demonstrate that unique patterns of TLR expression and novel signaling cascades create unique responses to microbial products by microglial, monocytic, and T cell lineages. In particular, TLR3-mediated HIV reactivation by IRF3 in microglial cells is a novel pathway, which allows HIV to emerge from latency in infected microglia and potentially cause neuronal damage.

The TLR pathways may also exacerbate neuronal damage in response to viral proteins and drugs of abuse. Recently, El-Hage et al. [108] have shown that exposure to HIV-1 Tat and/or gp120 altered TLR expression in astrocytes, providing a clue on how viral proteins may interfere with the innate immune response of the CNS to HIV-1. Similarly, Dutta et al. [109] showed that morphine and HIV Tat, together, can lead to up-regulation of TLR2, 4 and 9, enhanced pro-inflammatory cytokines (IL-6, TNF-α) levels, and neuronal damage.

Since HIV patients characteristically have chronic inflammation due to the release of microbial components into the circulation, TLR responses in each of these cell types is likely to contribute to disease progression. Manipulation of TLR signaling pathways is likely to find applications in strategies for viral eradication and/or silencing [110], since these receptors are differentially engaged in cells of the CNS, the monocyte/macrophage lineage, and T cell subsets.

## Methods

### Development of latently-infected cells

The human immortalized microglial cells (hµglia) used to generate HIV-latently infected cells are described in Garcia-Mesa et al. [40]. HIV infection was then conducted essentially as previously described for CHME-5/HIV [46] to obtain mixed and clonal populations of hµglia/HIV cells. Briefly, infection by spinoculation was carried out with vesicular stomatitis virus G-(VSVG) pseudotyped lentiviral vectors bearing a fragment of HIV-1_pNL4-3_, containing *Tat*, *Rev*, *Env*, *Vpu*, and *Nef* (some cell lines contain an older HIV construct carrying no *Nef* [44]) cloned into the pHR’ backbone together with the short-lived green fluorescence protein (d2EGFP), as previously shown [44, 111]; (Fig. 2a). mNF-κB HIVs, bearing a fragment carrying mutations in the NF-κB binding sites on the LTR region [29], were used as negative controls. The viral particles were produced by the triple transfection of 293T cells using lipofectamine, and the vector titer was determined as described previously [46, 112]. GFP^+^ cells (mixed and clonal populations) were isolated 48 h post-infection by fluorescence-activated cell sorting (FACS), further cultured, expanded, and allowed to enter into a latent state (stable, low GFP expression) for four weeks or more, depending on the cell type. hT-CHME-5 cells, described in Garcia-Mesa et al. [40], was used to obtain hT-CHME-5/HIV cells by superinfection with HIVs, as above. Routine evaluation of HIV latency was performed by treatment with TNF-α (Sigma-Aldrich, Cat. # T6674) or HDACi 4b [51], or any other appropriate stimulator for 16 h prior to quantification of GFP^+^ cells by flow cytometry analysis (see below). To keep HIV basal expression low (below 5%), latent cells were maintained in 1% FBS (in DMEM supplemented with 1× normocin) instead of 5% FBS.

For producing HIV-latently infected THP-1 (ATCC number: TIB-202), U937 (ATCC number: CRL-1593.2), and SC (ATCC number: CRL-9855) monocytic cell lines, uninfected cells were cultured on a 6-well plate at a density of (1 × 10^6^ cells per well) in RPMI growth medium containing 10% fetal bovine serum (FBS), 1% penicillin/streptomycin, and 50 nM of 2-mercaptoethanol for THP-1 and U937, or in Iscove’s modified Dulbecco’s medium with 4 mM l-glutamine adjusted to contain 1.5 g/L sodium bicarbonate and supplemented with 50 nM 2-mercaptoethanol, 0.1 mM hypoxanthine and 16 µM thymidine, and 10% FBS for SC cells. Infection was carried out by spinoculation, as described above. Latency of HIV provirus was characterized by treatment with TNF-α, LPS (Invivogen, Cat. # tlrl-peklps), or α-CD3/α-CD28 mAb (TCR) beads (Invitrogen, Dynabeads^®^ Human T-Activator CD3/CD28 11161D) for 16 h prior to quantification of GFP^+^ cells by FACS (flow cytometry) analysis. Jurkat/HIV (2D10) cells [44] was used as control.

### Integration site analysis

We basically followed the protocol described in Wu et al. [113] with some modifications. Briefly, we digested 250 ng of isolated genomic DNA (using the Qiagen DNeasy Blood and Tissue kit) with *MseI* and *BglII* Fast Digest restriction endonucleases (ThermoFisher Scientific) in Fast Digest buffer for 5 min at 37 °C, followed by heat-inactivation for degree for 10 min at 70 °C. 50 ng of the digest was then ligated with 100 ng of phosphorylated and annealed *MseI* linker using Quick Ligation (ThermoFisher Scientific) for 5 min at room temperature (per manufactures instructions), and then heat-inactivated for 10 min at 70 °C. The product was then subjected to a first PCR round using the Phusion Flash High Fidelity Taq master mix (ThermoFisher Scientific), and following a protocol consisting of initial denaturing at 98 °C for 10 sec (1 cycle), and denaturing at 95 °C for 1 s, annealing at 56 °C for 5 s and extension at 72 °C for 15 s (25 cycles). In the reaction mix (20 µL total volume), we added 10 µL of 2× Phusion Flash PCR Master mix, 1 µL of Fwd HIV-1 3′ LTR Primer (10 µM; AGTGCTTCAAGTAGTGTGTGCC), 1 µL of Rvs Linker Primer (10 µM; GTAATACGACTCACTATAGGGC), and 5 ng of ligated DNA. A second PCR round, using the same master mix, was carried out by initial denaturing at 98 °C for 10 s (1 cycle), and denaturing at 95 °C for 1 s, annealing at 60 °C for 5 s and extension at 72 °C for 15 s (25 cycles). In the reaction mix (50 µL total volume), we added 25 µL of 2× Phusion Flash PCR Master mix, 0.5 µL of Gex-Barcode-A Ion Adapter (50 µm), 1 µL of Fwd HIV-1 3′ LTR Primer, 1 µL of Rvs Linker Primer, and 1 µL of the first round PCR product. Finally, the second round PCR product was run on a 1.5% agarose gel and sizes from 200 to 350 were selected for sequencing. 300 pg of DNA was used for Ion Torrent sequencing following manufactures protocol, and the flanking sequences of genomic DNA were analyzed using the BLAT alignment tool (http://genome.ucsc.edu).

### Reagents, cell culture, and treatments

TLR ligands (Human TLR1–9 Agonist kit, Invivogen, Cat. # tlrl-kithw) were prepared as recommended by the manufacturer prior to addition to cell cultures. Safe, non-toxic doses of these TLR agonists were chosen by the propidium iodide (PI) exclusion method [described elsewhere, counting stained (dead) cells with a Cellometer^®^ Vision automatic cell counter (Nexcelom Bioscience, MA)], after experimentation with THP-1/HIV (HA3) cells for further treatment with the rest of the cell lines tested.

For testing NF-κB dependence of TLR ligands-mediated HIV reactivation, cells were pre-treated for 2 h with either 100 µM of IKKγ NEMO binding domain inhibitory peptide, or equivalent amount of the control peptide (Imgenex), prior to incubation with indicated doses of TLR ligands for 16 h. Quantification of GFP^+^ cells by flow cytometry followed.

In general, assays on suspension cells (THP-1, U937, SC, and Jurkat) were carried out at a density of 1 × 10^6^ cells per mL, in 96-well plates in a volume of 100 µL. Assays on microglial cells (attached) were carried out in 24-well plates containing 1 × 10^5^ cells per well plated at least 8 h prior to treatments. Cell culture maintenance was carried out at 37 °C in 5% CO_2_, and treatments were performed under the same conditions for 16 h prior to evaluation of viral reactivation by flow cytometry and/or fluorescence microscopy.

### Flow cytometry and microscopy

Quantification of GFP-expressing cells was carried out by fluorescence-activated cell sorting (FACS or flow cytometry) analysis using the LSRFortessa instrument for cell sorting, the FACSDiva software (BD, NJ) for data collection, and the WinList 3D software for data analysis. For counting GFP^+^ cells, treated and untreated cells were collected and resuspended in 300 µL of cold PBS.

Further characterization of the newly-developed hµglia/HIV cells included measuring the surface expression of CD11b (BD 553310), P2RY12 (Abcam ab86195) and CD14 (eBioscience 12-0149), as well as the expression of TLR3 (SCBT sc-10740) by fluorescence microscopy. For fluorescence microscopy, microglial cells were cultured on glass coverslips, treated, and subjected to immunofluorescence with anti-CD11b-FITC, anti-CD14-PE, anti-P2RY12-PE antibodies, or secondary PE-conjugated antibody for anti-TLR3 primary antibody to detect expression of the target receptors. Briefly, treated cells were washed, fixed with 4% paraformaldehyde, and permeabilized with 0.1% Triton X-100 prior to incubation with antibodies for 2 h followed by washing with DAPI-containing washing solution for nuclear staining. For FACS analysis, used to evaluate surface expression of TLR1 (SCBT sc-130896), TLR2 (BD 558318), TLR3 (SCBT sc-10740), TLR4 (eBioscience 12-9917), TLR5 (SCBT sc-130897), TLR6 (SCBT sc-30001), TLR7 (SCBT sc-30004), TLR8 (SCBT sc-25467), or TLR9 (SCBT sc-25468), we used 1 × 10^5^ cells resuspended in 1 mL of cold PBS in the presence of 0.5 µg of the antibody or equivalent amount of species-specific isotype control antibody for 20 min on ice. For comparative profile of TLR expression, we used serum-starved HIV-infected or uninfected microglia, monocytic, and T cells, in the absence or presence of poly (I:C) (100 ng/mL). TLR binding to cognate antibody was carried out as described above. Appropriate secondary antibodies were used in the absence of fluorophore-conjugated primary antibody. Cell-antibody complexes were centrifuged, and the pellet resuspended in 300 µL of PBS before FACS analysis.

To perform experiments to test TLR3-mediated HIV reactivation through pharmacological inhibition, we purchased LY294002 (Sigma-Aldrich, L2908), bufalin (Sigma-Aldrich, B0261), and wortmannin (Sigma-Aldrich, W1628). These compounds were prepared as indicated by the manufacturers.

Where applicable, cells were brightfield-photographed and imaged for GFP^+^ fluorescence emission with a Nikon TE2000 inverted scope equipped with a DS-QiMc camera and controlled by NIS Elements software (Nikon). Images were produced by using the ImageJ software (NIH).

### Detection of TLR stimulation in THP1-XBlue™ cells

THP1-XBlue™ cells (Invivogen, Cat. # thpx-sp) were cultured as recommended by the *Invivogen* protocol. After the treatment with the TLR ligands or pre-treatment with the CDK inhibitors DRB (10 µM) or flavopiridol (30 nM) prior to treatment with TLR ligands, at the doses indicated in the Fig. legend, cells were centrifuged at 1500 rpm for 5 min, while preparing the QUANTI-Blue™ following the instructions on the pouch (Invivogen). 180 µL of resuspended QUANTI-Blue™ was mixed with 20 µL of cell supernatant in a well of a flat-bottom 96-well plate, and incubated at 37 °C for the periods of time indicated (for 4 h in the experiments involving the inhibitors) prior to determining the SEAP levels using a spectrophotometer at 620–655 nm.

### SDS-PAGE/Western blot analysis

Sodium dodecyl sulfate polyacrylamide gel electrophoresis (SDS-PAGE)/Western blot analysis (carried out as described elsewhere) of NF-κB p65 or IRF3 was used to assess pathway activation of NF-κB or IRF3, respectively. Briefly, cells were treated with TNF-α or shown TLR ligand at the indicated concentration for the indicated time period. For this, 7 × 10^6^ cells per treatment were cultured or plated (microglia), and treated as described above. After two washings with cold PBS, cells were collected in 500 µL of Buffer A [10 mM Hepes/KOH, pH 7.9, 1.5 mM MgCl_2_, 10 mM KCl, 1 mM ethylenediaminetetraacetic acid (EDTA)] in the presence of phenylmethylsulfonyl fluoride (PMSF; 1 mM), dithiothreitol (DTT; 1 mM), 0.5% nonyl phenoxypolyethoxylethanol (NP-40), and 1× Halt^®^ protease/phosphatase inhibitors cocktail (Pierce), and cytoplasmic extracts were recovered in the supernatant after centrifugation at 1500×*g* for 10 min at 4 °C. Nuclei were then washed three times in Buffer A, and re-suspended in 100 µL of Buffer B (20 mM Hepes/KOH, pH 7.9, 25% glycerol, 420 mM NaCl, 1 mM EDTA, 1.5 mM MgCl_2_) containing PMSF (1 mM), DTT (1 mM) and the inhibitors cocktail, and centrifuged at 20,000×*g* for 15 min at 4 °C to recover nuclear extracts (NE). Protein concentration in NE was measured by Bradford assay, and protein solutions were subjected to SDS-PAGE/Western blot using the Santa Cruz Biotechnology antibodies against NF-κB p65 (sc-372), IRF3 (BD Biosciences #550428), or SPT-5 (sc-28678) or TBP (sc-273), as loading controls. These primary antibodies were bound by the appropriate IRDye 800CW or 680LT secondary antibody, and the membranes were scanned and analyzed using the Odyssey^®^ Infrared Imaging System (LI-COR Biosciences, NE). Similarly, expression of CycT1 and CDK9 in whole cell extracts (WCE) from untreated or treated hµglia/HIV (HC01) and (HC69) cells was detected by SDS-PAGE/Western blot using anti-CycT1 antibody (SCBT sc-10750) or anti-CDK9 antibody (SCBT sc-8338), respectively, and anti-tubulin antibody as loading control.

### Chromatin immunoprecipitation (ChIP)

Sample preparation for ChIP experiments were carried out essentially as previously described [44, 61] with minor modifications. For each experimental condition, 7 × 10^6^ HC69 cells were plated on a 150-mm diameter plate and incubated overnight. Cells were then left untreated or treated with either TNF-α (10 ng/mL), poly (I:C) (100 ng/mL) or LPS (10 ng/mL) for 30 min. The cells were then cross-linked in 1% formaldehyde, incubated for 10 min at ambient temperature, and the reaction quenched by adding glycine 1 mM, and further incubated for 5 min. After a double wash with cold phosphate buffer saline (PBS), cells were collected in PBS/Halt^®^ cocktail solution by centrifugation for 5 min at 3000×*g*. The cell pellets were resuspended in 500 µL of CE buffer vortexed, and incubated for 10 min on ice. Nuclei were collected by centrifugation for 3 min at 9000×*g*, and resuspended 250 µL of SDS lysis buffer(1% SDS, 10 mM EDTA, 50 mM Tris–HCl, pH 8.1), incubated on ice for 15 min with periodic vortexing, and sonicated in a water bath using a Biorupter Plus water bath sonicator for 20 min (30″ ON/30″ OFF cycles). Under these conditions the chromatin was cleaved to between 100 and 500 bp.

Fragmented chromatin was recovered in the supernatant after centrifugation for 5 min at 9000×*g*. 5 µg of control IgG or anti-RNAP II pSer5 (ab5131, Abcam), anti-NF-κB p65 (C-20, SCB), or anti-IRF3 (BD Biosciences #550428) antibodies were incubated in a blocked protein A/G coated plate for 30 min. For each immunoprecipitation, 45 µL of the chromatin fractions were diluted in 100 µL of IP dilution buffer and added to antibody coated well. Antibody binding reactions were carried out for 1 h at ambient temperature with 500 rpm shaking. After two washes with RIPA buffer (25 mM Tris, pH 7–8, 150 mM Na, 0.1% SDS, 0.5% sodium deoxycholate, 1% Triton X-100) and one wash with TE buffer, chromatin-IgG complexes were eluted and digested in elution/Proteinase K buffer for 30 min at 65 °C. Freed DNA was purified with PCR magnetic clean up beads (PCR cleanup beads, Axygen).

### Chromatin immunoprecipitation library preparation, enrichment and sequencing

Following purification, the ChIP DNA was end repaired (daTailed). Reactions contained 1× ligase buffer (NEB), 1 mM dNTPs (NEB), 6 units of T4 DNA polymerase (NEB), 2 units or Klenow (NEB), 20 units of T4 Polynucleotide kinase (NEB) and 1.25 units of Taq DNA polymerase (NEB) and incubated at 20° for 30 min followed by 65 °C for an additional 30 min. MiFWD (Sense; TCGTCGGCAGCGTCT), (Antisense; GACGCTGCCGACGA) and MiRVS (Sense; GTCTCGTGGGCTCGGT), (Antisense; CCGAGCCCACGAGAC) adapters were ligated (T4 quick ligase, ThermoFisher) on to the end repaired DNA and Ioncode barcodes were added to each sample using 35 cycle PCR.

Samples were pooled and HIV was enriched using hybridization to biotinylated HIV probes. The probes were produced by sonicating the HIV-1_pNL4-3_ construct to generate 500 bp fragments. The probes were then end repairing/daTailed using the protocol as described above and Biotin-14-dATP was added using terminal transferase. To enrich for HIV sequences, the pooled barcoded library was added to 6× SSC buffer, 500 nM blocking oligos (pool of all barcodes), 5 µg Cot-1 DNA and water to bring up to 98 µL and incubated for 10 min at 95 °C. Immediately after, 100 ng of biotinylated HIV probes were added to the sample, vortexed, and the temperature was reduced to 65 °C and incubated for 1 h. After hybridization, the biotinylated probe/HIV library was bound to 13 µL of washed myOne streptavidin Dynabeads (Thermofisher). Beads were then pelleted on a magnet and washed 3 times with 200 µL of Ion Torrent wash buffer. Each wash was performed by pipetting up and down 10 times followed by vortexing for 10 s. The last wash was done with 200 µL of ddH_2_O and then the beads were re-suspended in 10 µL of fresh ddH_2_O.

The DNA on the beads were amplified using a BesTaq master mix (ABMgood) and primers to the Ion Torrent adapters A and trP1 for 35 cycles. The library was size selected between 200 and 500 bp using gel electrophoresis followed by 1:4 water diluted PCR clean up bead isolation. The enriched library was sequenced using the S5 Torrent Sequencer using a 540 chip following the manufactures protocol.

Following sequencing, the data were analyzed using the Geneious suite of software. Each sample was separated based on the Ioncode barcodes, adapters were trimmed and each sequence was mapped to a 3′ LTR deleted HIV-1_pNL4-3_ genome.

### Generation of *Mycobacterium tuberculosis* (Mtb)-derived compounds

Mtb (H37Rv) fractions and glycolipids from the TBVTRM Collection (NIAID, HHSN266200400091c contract) were provided by the BEI Resources (Manassas, VA). Cell wall-associated glycolipids were: phosphatidylinositol mannosides (PIM_1_,_2_, NR-14846; PIM_6_, NR-14847), lipomannan (LM, NR-14850) and lipoarabinomannan (LAM, NR-14848). Full-length LprG (rLprG, Rv1411c) was amplified from Mtb H37Rv genomic DNA by PCR, cloned in *E. coli* and expressed in *M. smegmatis* as previously described [114]. Treatment of cells with Mtb-derived compounds was carried out for 16 h prior to evaluation of HIV reactivation.

## Additional files

**Additional file 1: Fig. S1.** HIV emerges from latency in monocytic cells. TNF-α- and LPS-mediated reactivation of HIV in latently-infected THP-1/HIV (HA3), U937/HIV (HUC5), and SC/HIV (HSCC4) monocytic cells. Cells treated with TNF-α (10 ng/mL) or LPS (1 µg/mL) were subjected to flow cytometry (FACS) analysis 16 h post-treatment initiation. As in the main Figs., in the FACS profiles GFP^+^ cell populations are shown in bright green, and the % of GFP-expressing cells is indicated. TCR-mediated reactivation as well as Jurkat/HIV 2D10 cells [44] were used as control.

**Additional file 2: Fig. S2.** HIV reactivation by TLR agonists in latently-infected microglial cells: Treatment of human hµglia/HIV (HC01) and rat hT-CHME-5/HIV (HC03) clonal populations with TLR ligands. Cells were plated 8 h before no treatment or treatment with TLR agonists Pam3CSK4 (1 µg/mL), poly (I:C) (10 µg/mL), LPS (5 µg/mL), flagellin (5 µg/mL) or PIM_6_ (5 µg/mL) for 16 h prior to measuring GFP expression by FACS analysis.

**Additional file 3: Fig. S3.** Compiled data for the relative induction (Y-axis) of Mtb TLR2 ligands and HDAC inhibitor (SAHA or HDACi 4b) (X-axis). For indicated microglial cells (**a**) and monocytes (**b**), the data was TNF-α-normalized. For indicated T cells (**c**), the data was α-CD3/CD28-normalized. **a** Microglial cells are represented by hµglia/HIV (HC01; black bars), hµglia/HIV (HC69; red bars), and hT-CHME-5/HIV (HC14; blue bars). **b** The monocytic cells are represented by THP-1/HIV (HA3; black bars), U937/HIV (HUC5; red bars), and SC/HIV (HSCC4; blue bars). **c** T cells are represented by Jurkat/HIV (2D10; black bars) and Th17/HIV (mixed population; red bars). Error bars indicate three or more experiments.

**Additional file 4: Fig. S4.** TLR2 and TLR4 are expressed on monocytic cells. Flow cytometry analysis of TLR2 (left column) and TLR4 (right column) surface expression on THP-1/HIV (HA3), U937/HIV (HUC5), and SC/HIV (HSCC4) cells. Cells were incubated with anti-TLR2-Alexa Fluor (red), anti-TLR4-PE (blue), or isotype control (grey) antibodies prior to FACS analysis. Fraction of cells expressing TLR is depicted in % in the flow cytometry profiles.

**Additional file 5: Fig. S5.** HIV reactivation is impaired in CHME-5 and THP-1 cells latently-infected with viruses carrying mutant NF-κB binding sites. HIV expression was induced in **a** CHME-5/HIV (H1F3) and CHME-5/HIV_mNF-κB (mixed population), and **b** THP-1/HIV (HA3) and THP-1/HIV_mNF-κB (mixed population) cells by flagellin (5 µg/mL for microglia and 1 µg/mL for THP-1) or HDACi 4b (30 µM). Cells were incubated with activators for 16 h prior to measuring GFP-expressing cells by FACS. Fraction of cells expressing GFP is shown in %.

**Additional file 6: Fig. S6.** TLR-mediated HIV reactivation involves the NF-κB pathway and P-TEFb. **a** Treatment of the reporter THP1-XBlue™ cells with TLR ligands. THP1-XBlue™ cells were untreated or incubated (X-axis) with Pam3CSK4 (0.1 µg/mL), HKLM (10^8^ cells/mL), poly (I:C) (10 µg/mL), poly (I:C)_LMW (10 µg/mL), LPS (1 µg/mL), flagellin (1 µg/mL), FSL-1 (1 µg/mL), imiquimod (10 µg/mL), ssRNA40 (5 µg/mL), or ODN2006 (5 µM) for 2 (black), 4 (red) or 6 (blue) hours prior to quantification of SEAP released into the supernatant upon reaction with the QUANTI-Blue^®^ reagent by spectrophotometry at 620 nm (Optical Density; Y-axis). **b** Inhibition of TLR-mediated NF-κB activation. THP1-XBlue™ cells were untreated (black) or pre-treated with DRB (red; 10 µM) or flavopiridol (blue; 30 nM) for 30 min prior to treatment with Pam3CSK4 (0.1 µg/mL), HKLM (10^8^ cells/mL), poly (I:C) (10 µg/mL), poly (I:C)_LMW (10 µg/mL), LPS (1 µg/mL), flagellin (1 µg/mL), FSL-1 (1 µg/mL), imiquimod (10 µg/mL), ssRNA40 (5 µg/mL), and ODN2006 (5 µM), as shown in the X-axis, prior to quantification of SEAP (Y-axis). **c** Nuclear recruitment of NF-κB p65 in THP-1/HIV (HA3) cells treated with TNF-α or TLR ligands at doses indicated in **a** or **b** above. Representative Western blot analysis with anti-NF-κB p65 antibody (anti-SPT5 antibody used as loading control) of THP-1/HIV (HA3) cells nuclear extracts purified from cells treated for 30 min prior to NE purification. Molecular weight markers are shown in kDa.

**Additional file 7: Fig. S7.** TLR and PTEF-b inhibition impairs TLR-mediated HIV reactivation. **a** TLR ligands reactivate HIV in an NF-κB-dependent manner. Treatment of THP-1/HIV (HA3) cells with TNF-α (10 ng/mL) or TLR ligands (Pam3CSK4 at 0.1 µg/mL, HKLM at 10^8^ cells/mL, poly (I:C) at 10 µg/mL, LPS at 1 µ/mL, flagellin at 1 µ/mL, FSL-1 at 1 µg/mL, imiquimod at 10 µg/mL, ssRNA40 at 5 µg/mL, and ODN2006 at 5 µM) for 16 h after a 2-h pre-incubation with either 100 µM of IKKγ NEMO binding domain inhibitory peptide (red bars; Inh Pep) or equivalent amount of the control peptide (blue bars; Imgenex) (X-axis). Y-axis represents % of GFP-expressing cells after FACS measurements and blue squares % of viable cells after PI exclusion quantification (right Y-axis). Error bars depict the standard deviation of three different experiments. **b** Partial inhibition of TNF-α-, IL-1β-, or TLR-mediated HIV reactivation by P-TEFb inhibitors. Human hµglia/HIV (HC01) and (HC69), and rat hT-CHME-5/HIV (HC03) and (HC14) microglial cells were untreated (black) or pre-treated with DRB (red; 10 µM) or flavopiridol (blue; 30 nM) for 30 min prior to treatment with TNF-α (30 ng/mL), IL-1β (10 pg/mL), LPS (1 µg/mL), or poly (I:C) (10 µg/mL), as shown in the X-axis, for 16 h prior to quantification of GFP (Y-axis).

**Additional file 8: Fig. S8.** IL-1β, but not IL-6 or -8, reactivates HIV in latently infected microglial cells. hµglia/HIV (HC01) (black bars) and HC69 (red bars), CHME-5/HIV (H1F3) (blue bars), and hT-CHME-5/HIV (HC03) (pink bars) and HC14 (green bars) cells were incubated in the absence (Untreated) or presence of IL-1β (5 pg/mL), IL-6 (5 µg/mL) or IL-8 (5 µg/mL), as indicated in the X-axis, for 16 h prior to measuring GFP expression by flow cytometry, indicated in the Y-axis. Error bars represent standard deviation of three different experiments.

## Abbreviations

HIV: human immuno-deficiency virus
TLRs: toll-like receptors
CNS: central nervous system
HAND: HIV-associated neurocognitive disorders
GFP: green fluorescence protein
Mtb: *Mycobacterium tuberculosis*
hµglia/HIV: human immortalized microglial cells latently-infected with HIV
SV40Tag: simian virus 40 T antigen
LPS: lipopolysaccharide
NF-κB: nuclear factor kappa-light-chain-enhancer of activated B cells
HAART: highly-active anti-retroviral therapy
hTERT: human telomerase reverse transcriptase
LTR: long terminal repeats
TNF-α: tissue necrosis factor α
TCR: T cell receptor
PCR: polymerase chain reaction
HDAC: histone deacetylase
PI: propidium iodide
SDS-PAGE: sodium dodecyl sulfate polyacrylamide gel electrophoresis
WCE: whole cell extracts
NBD: NEMO binding domain
IL: interleukin
LM: lipomannan
ManLAM: mannosylated lipoarabinomannan
PIM: phosphatidylinositol mannoside
ChIP: chromatin immunoprecipitation
MNase: micrococcal nuclease
