# Supplementary figures and images for: Toll-like receptor 3 activation selectively reverses HIV latency in microglial cells
Source: Retrovirology. 2017 Feb 6;14:9. doi: 10.1186/s12977-017-0335-8 (PMC5294768; doi:10.1186/s12977-017-0335-8)

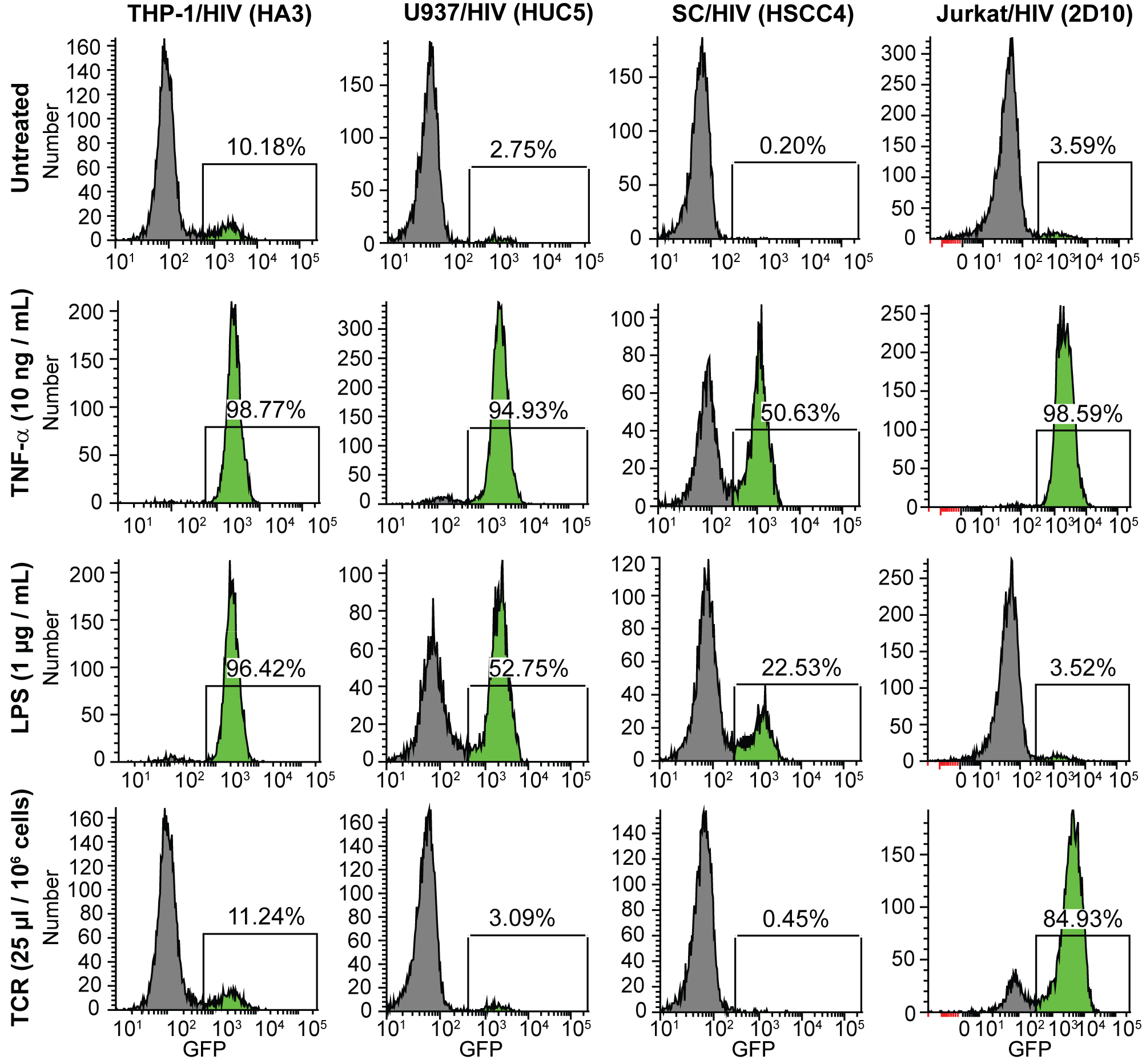

Supplement: Supplementary file 1 — Additional file 1: Fig. S1. HIV emerges from latency in monocytic cells. TNF-α- and LPS-mediated reactivation of HIV in latently-infected THP-1/HIV (HA3), U937/HIV (HUC5), and SC/HIV (HSCC4) monocytic cells. Cells treated with TNF-α (10 ng/mL) or LPS (1 µg/mL) were subjected to flow cytometry (FACS) analysis 16 h post-treatment initiation. As in the main Figs., in the FACS profiles GFP+ cell populations are shown in bright green, and the % of GFP-expressing cells is indicated. TCR-mediated reactivation as well as Jurkat/HIV 2D10 cells [44] were used as control. [file 12977_2017_335_MOESM1_ESM.tif]

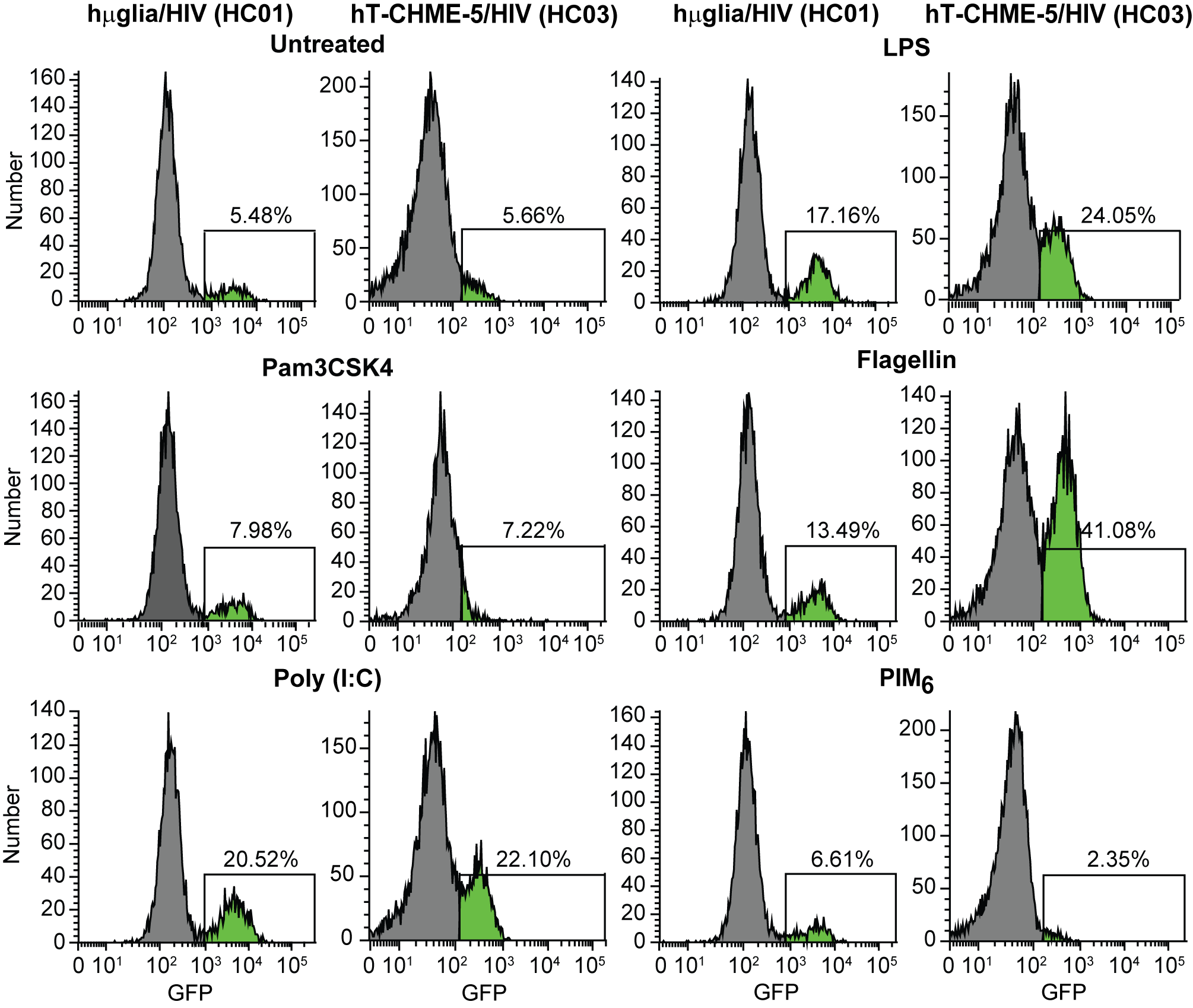

Supplement: Supplementary file 2 — Additional file 2: Fig. S2. HIV reactivation by TLR agonists in latently-infected microglial cells: Treatment of human hµglia/HIV (HC01) and rat hT-CHME-5/HIV (HC03) clonal populations with TLR ligands. Cells were plated 8 h before no treatment or treatment with TLR agonists Pam3CSK4 (1 µg/mL), poly (I:C) (10 µg/mL), LPS (5 µg/mL), flagellin (5 µg/mL) or PIM6 (5 µg/mL) for 16 h prior to measuring GFP expression by FACS analysis. [file 12977_2017_335_MOESM2_ESM.tif]

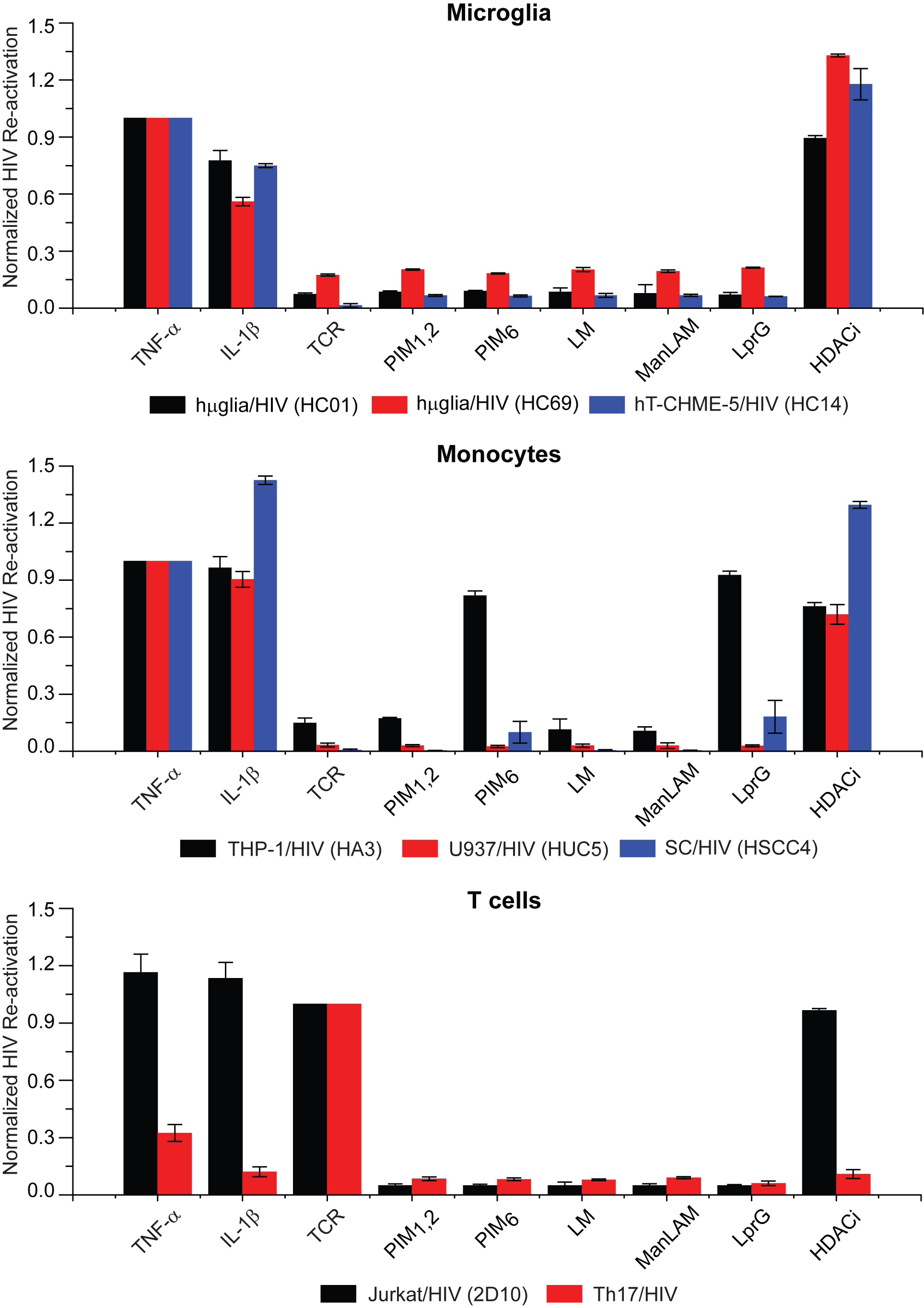

Supplement: Supplementary file 3 — Additional file 3: Fig. S3. Compiled data for the relative induction (Y-axis) of Mtb TLR2 ligands and HDAC inhibitor (SAHA or HDACi 4b) (X-axis). For indicated microglial cells (a) and monocytes (b), the data was TNF-α-normalized. For indicated T cells (c), the data was α-CD3/CD28-normalized. a Microglial cells are represented by hµglia/HIV (HC01; black bars), hµglia/HIV (HC69; red bars), and hT-CHME-5/HIV (HC14; blue bars). b The monocytic cells are represented by THP-1/HIV (HA3; black bars), U937/HIV (HUC5; red bars), and SC/HIV (HSCC4; blue bars). c T cells are represented by Jurkat/HIV (2D10; black bars) and Th17/HIV (mixed population; red bars). Error bars indicate three or more experiments. [file 12977_2017_335_MOESM3_ESM.tif]

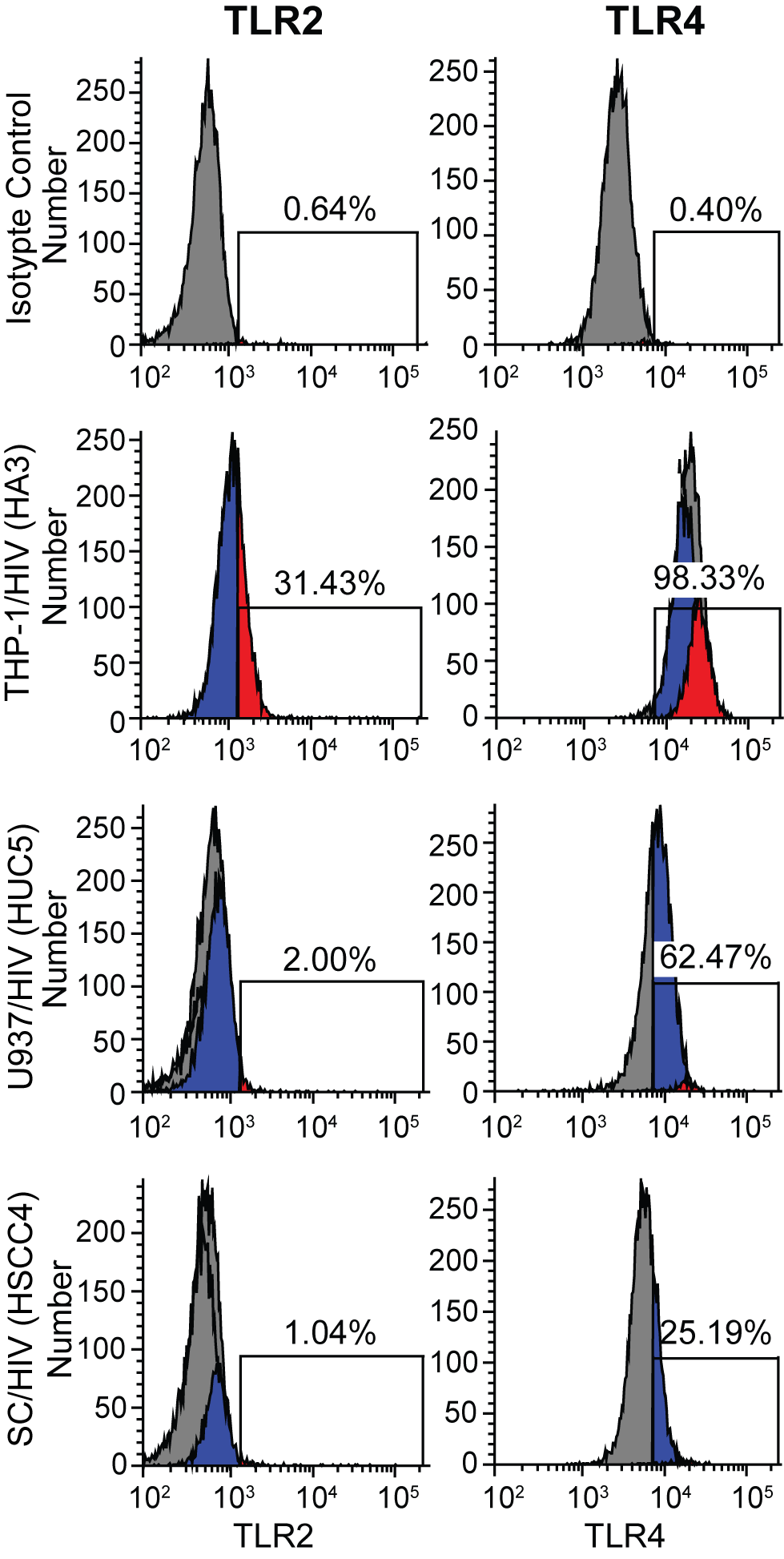

Supplement: Supplementary file 4 — Additional file 4: Fig. S4. TLR2 and TLR4 are expressed on monocytic cells. Flow cytometry analysis of TLR2 (left column) and TLR4 (right column) surface expression on THP-1/HIV (HA3), U937/HIV (HUC5), and SC/HIV (HSCC4) cells. Cells were incubated with anti-TLR2-Alexa Fluor (red), anti-TLR4-PE (blue), or isotype control (grey) antibodies prior to FACS analysis. Fraction of cells expressing TLR is depicted in % in the flow cytometry profiles. [file 12977_2017_335_MOESM4_ESM.tif]

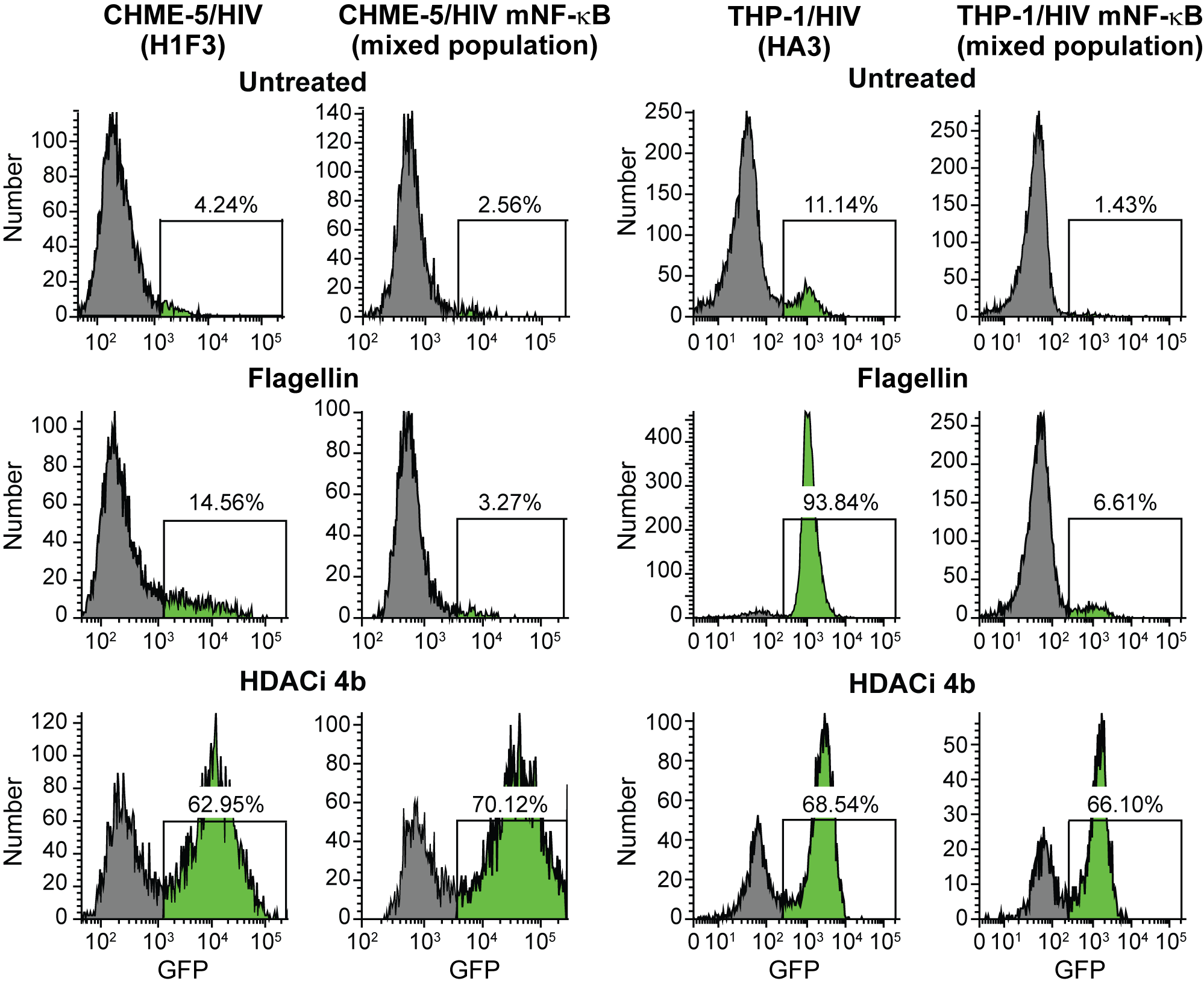

Supplement: Supplementary file 5 — Additional file 5: Fig. S5. HIV reactivation is impaired in CHME-5 and THP-1 cells latently-infected with viruses carrying mutant NF-κB binding sites. HIV expression was induced in a CHME-5/HIV (H1F3) and CHME-5/HIV_mNF-κB (mixed population), and b THP-1/HIV (HA3) and THP-1/HIV_mNF-κB (mixed population) cells by flagellin (5 µg/mL for microglia and 1 µg/mL for THP-1) or HDACi 4b (30 µM). Cells were incubated with activators for 16 h prior to measuring GFP-expressing cells by FACS. Fraction of cells expressing GFP is shown in %. [file 12977_2017_335_MOESM5_ESM.tif]

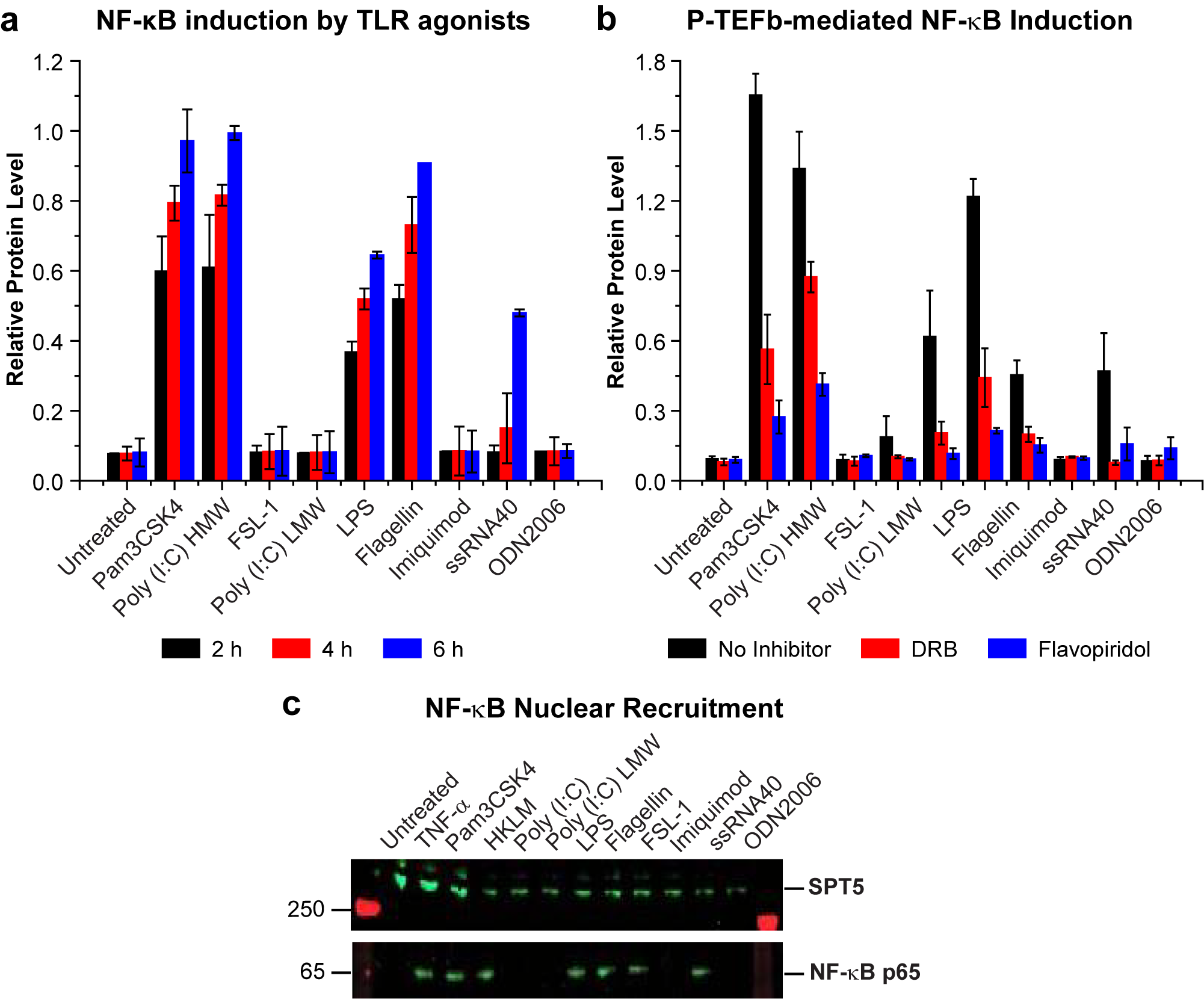

Supplement: Supplementary file 6 — Additional file 6: Fig. S6. TLR-mediated HIV reactivation involves the NF-κB pathway and P-TEFb. a Treatment of the reporter THP1-XBlue™ cells with TLR ligands. THP1-XBlue™ cells were untreated or incubated (X-axis) with Pam3CSK4 (0.1 µg/mL), HKLM (108 cells/mL), poly (I:C) (10 µg/mL), poly (I:C)_LMW (10 µg/mL), LPS (1 µg/mL), flagellin (1 µg/mL), FSL-1 (1 µg/mL), imiquimod (10 µg/mL), ssRNA40 (5 µg/mL), or ODN2006 (5 µM) for 2 (black), 4 (red) or 6 (blue) hours prior to quantification of SEAP released into the supernatant upon reaction with the QUANTI-Blue® reagent by spectrophotometry at 620 nm (Optical Density; Y-axis). b Inhibition of TLR-mediated NF-κB activation. THP1-XBlue™ cells were untreated (black) or pre-treated with DRB (red; 10 µM) or flavopiridol (blue; 30 nM) for 30 min prior to treatment with Pam3CSK4 (0.1 µg/mL), HKLM (108 cells/mL), poly (I:C) (10 µg/mL), poly (I:C)_LMW (10 µg/mL), LPS (1 µg/mL), flagellin (1 µg/mL), FSL-1 (1 µg/mL), imiquimod (10 µg/mL), ssRNA40 (5 µg/mL), and ODN2006 (5 µM), as shown in the X-axis, prior to quantification of SEAP (Y-axis). c Nuclear recruitment of NF-κB p65 in THP-1/HIV (HA3) cells treated with TNF-α or TLR ligands at doses indicated in a or b above. Representative Western blot analysis with anti-NF-κB p65 antibody (anti-SPT5 antibody used as loading control) of THP-1/HIV (HA3) cells nuclear extracts purified from cells treated for 30 min prior to NE purification. Molecular weight markers are shown in kDa. [file 12977_2017_335_MOESM6_ESM.tif]

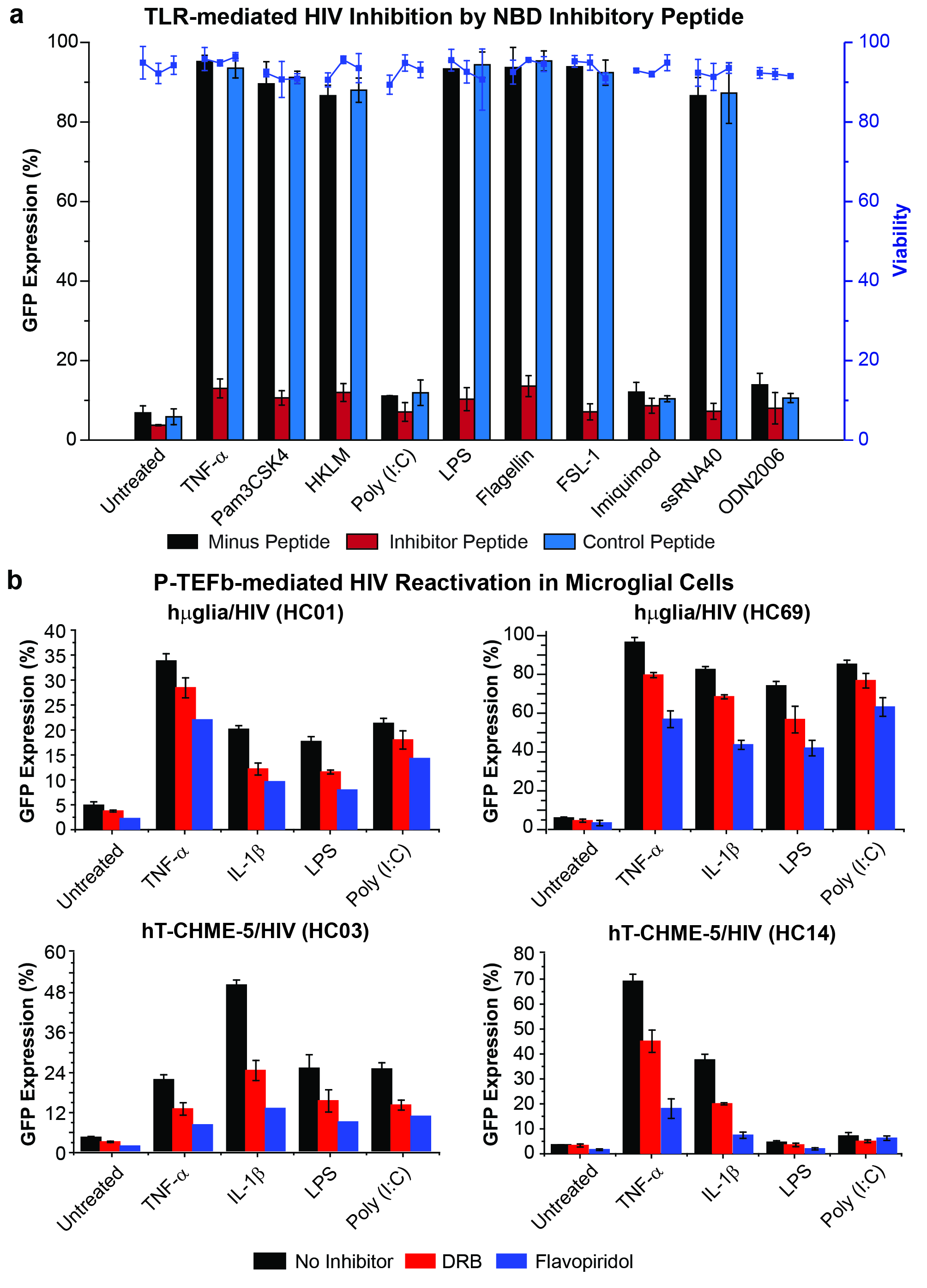

Supplement: Supplementary file 7 — Additional file 7: Fig. S7. TLR and PTEF-b inhibition impairs TLR-mediated HIV reactivation. a TLR ligands reactivate HIV in an NF-κB-dependent manner. Treatment of THP-1/HIV (HA3) cells with TNF-α (10 ng/mL) or TLR ligands (Pam3CSK4 at 0.1 µg/mL, HKLM at 108 cells/mL, poly (I:C) at 10 µg/mL, LPS at 1 µ/mL, flagellin at 1 µ/mL, FSL-1 at 1 µg/mL, imiquimod at 10 µg/mL, ssRNA40 at 5 µg/mL, and ODN2006 at 5 µM) for 16 h after a 2-h pre-incubation with either 100 µM of IKKγ NEMO binding domain inhibitory peptide (red bars; Inh Pep) or equivalent amount of the control peptide (blue bars; Imgenex) (X-axis). Y-axis represents % of GFP-expressing cells after FACS measurements and blue squares % of viable cells after PI exclusion quantification (right Y-axis). Error bars depict the standard deviation of three different experiments. b Partial inhibition of TNF-α-, IL-1β-, or TLR-mediated HIV reactivation by P-TEFb inhibitors. Human hµglia/HIV (HC01) and (HC69), and rat hT-CHME-5/HIV (HC03) and (HC14) microglial cells were untreated (black) or pre-treated with DRB (red; 10 µM) or flavopiridol (blue; 30 nM) for 30 min prior to treatment with TNF-α (30 ng/mL), IL-1β (10 pg/mL), LPS (1 µg/mL), or poly (I:C) (10 µg/mL), as shown in the X-axis, for 16 h prior to quantification of GFP (Y-axis). [file 12977_2017_335_MOESM7_ESM.tif]

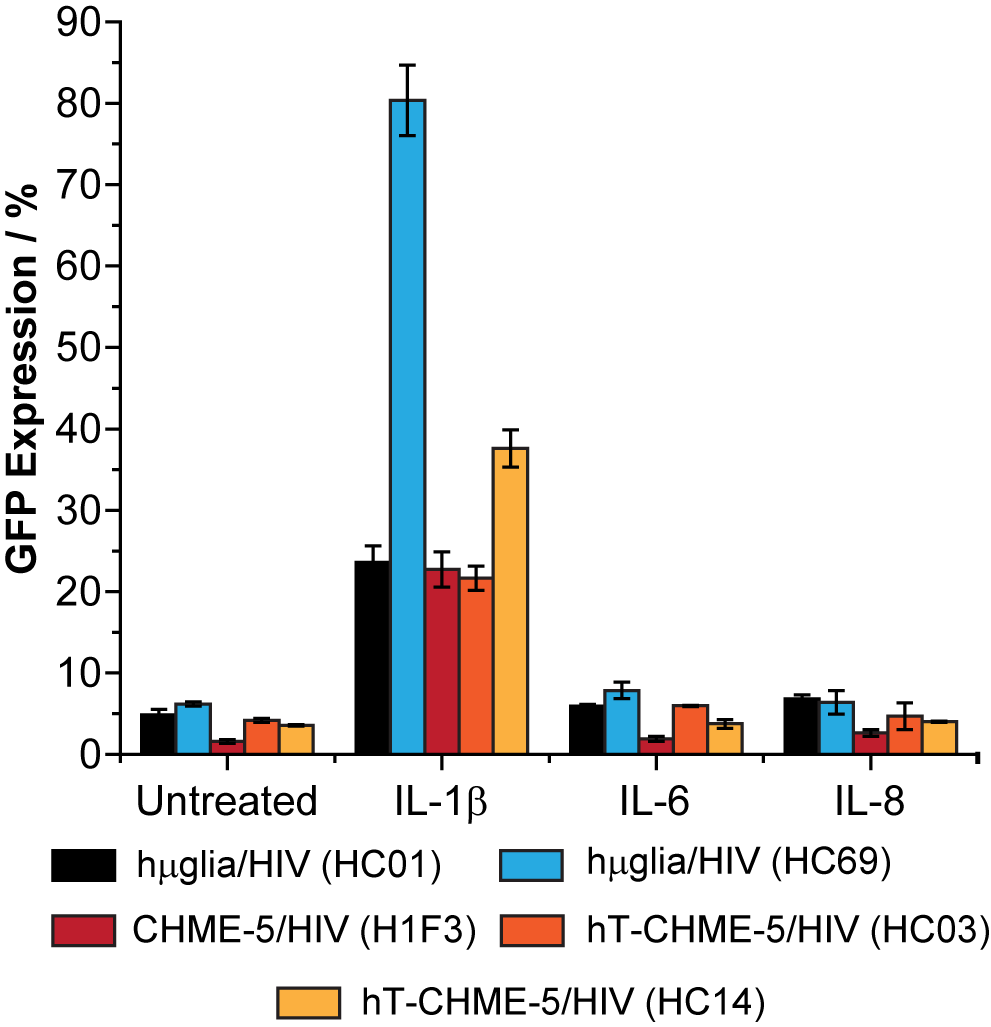

Supplement: Supplementary file 8 — Additional file 8: Fig. S8. IL-1β, but not IL-6 or -8, reactivates HIV in latently infected microglial cells. hµglia/HIV (HC01) (black bars) and HC69 (red bars), CHME-5/HIV (H1F3) (blue bars), and hT-CHME-5/HIV (HC03) (pink bars) and HC14 (green bars) cells were incubated in the absence (Untreated) or presence of IL-1β (5 pg/mL), IL-6 (5 µg/mL) or IL-8 (5 µg/mL), as indicated in the X-axis, for 16 h prior to measuring GFP expression by flow cytometry, indicated in the Y-axis. Error bars represent standard deviation of three different experiments. [file 12977_2017_335_MOESM8_ESM.tif]
